# Association Between Cannabis Use and Neuropsychiatric Disorders: A Two-sample Mendelian Randomization Study

**DOI:** 10.31083/AP46108

**Published:** 2025-08-28

**Authors:** Wei Guo, Lin Dong, Qingxing Lu, Mengtong Xie, Yuqi Yang, Yanchi Zhang, Xiaoyu Lu, Qiong Yu

**Affiliations:** ^1^Jilin Provincial Center for Disease Control and Prevention, 130062 Changchun, Jilin, China; ^2^Department of Epidemiology and Biostatistics, School of Public Health, Jilin University, 130021 Changchun, Jilin, China; ^3^Department of Psychology, Changchun Sixth Hospital, 130052 Changchun, Jilin, China; ^4^Innovative Biotechnology Laboratory, Jilin Biological Research Institute, 130012 Changchun, Jilin, China

**Keywords:** GWAS summary statistics, single nucleotide polymorphism, lifetime cannabis use, neuropsychiatric disorders

## Abstract

**Background:**

The progressive legalization and widespread use of cannabis has led to its use as a treatment for certain neuropsychiatric disorders. Traditional epidemiological studies suggest that cannabis use has an effect on some neurocognitive aspects. However, it is unclear whether cannabis use is causally related to common neuropsychiatric disorders. The present study was conducted to illustrate the causal relationships of genetically predicted cannabis use with common neuropsychiatric disorders.

**Methods:**

We used a two-sample Mendelian randomization method using genome-wide association study (GWAS) summary statistics obtained from publicly available databases on lifetime cannabis use and 10 neuropsychiatric disorders, including multiple sclerosis (MS), Alzheimer’s disease (AD), amyotrophic lateral sclerosis (ALS), autism spectrum disorder (ASD), epilepsy, generalized epilepsy, focal epilepsy, migraine, migraine with aura, migraine without aura, schizophrenia (SCZ), anorexia nervosa (AN), attention-deficit/hyperactivity disorder (ADHD), and Parkinson’s disease (PD) were studied with a two-sample Mendelian randomization method for GWAS summary statistics. The inverse variance weighted (IVW) method was used as the main analysis model.

**Results:**

Our study suggests that lifetime cannabis use is associated with an increased risk of developing PD (odds ratio (OR) = 1.782; 95% CI 1.032–3.075; *p* = 0.038) and an increased risk of ADHD in female participants (OR = 1.650; 95% CI 1.051–2.590; *p* = 0.029).

**Conclusions:**

Cannabis intake may cause adverse effects relating to certain neuropsychiatric disorders. Therefore, special attention should be paid to the side effects of addictive drugs during clinical treatment to avoid harmful effects on the brain and neurocognition.

## Main Points

1. This study is the first to explore the causal relationship between lifetime cannabis use and a variety of common neuropsychiatric disorders. Mendelian randomization studies, as an emerging epidemiological research methodology that teaches observational studies clear advantages in avoiding confounders and causality exploration, provide relevant information in studies on cannabis use to date.

2. This study included ten common neuropsychiatric disorders and provides a more definitive risk assessment for the exploration of the etiology of cannabis use in neuropsychiatric disorders.

3. Lifetime cannabis use was causally associated with Parkinson’s disease and the development of attention-deficit/hyperactivity disorder (ADHD) in female participants.

## 1. Introduction

In recent years, a growing number of studies have shown that on a global scale, some common of neurological disorders diseases such as Alzheimer’s disease(AD) and migraine are among the top ranking diseases, with their incidence rates continuing to rise [1, 2]. As the global population ages, neuropsychiatric diseases have threatened human health and increased the burden of disease. The global prevalence of cannabis use has increased due to its legalization in several regions [3]. Cannabis, the world’s most used drug and the next most popular psychoactive product after tobacco and alcohol, is increasing in use and includes more than 100 cannabinoids that interact with the body’s endocannabinoid system, which is made up of neurotransmitters such as Anandamide, which interact with cannabinoid receptors (CBRs) throughout the central nervous system (CNS) that bind to them and are associated with the development of common neurological disorders [4, 5]. There is growing evidence of a link between cannabis use and neuropsychiatric disorders. There is an effect of cannabis use on the development of psychotic symptoms, with a doubling of the risk of disease in susceptible individuals [6].

A study analysed that cannabis can affect the central nervous system by interacting with the endocannabinoid system (ECS) in the human body, which in turn has an effect on the central nervous system [7], which is strongly associated with the development of several psychiatric disorders, neurodegenerative diseases and movement conditions [8]. In a prospective study on clinical, genetic and environmental risk factors for multiple sclerosis, patients with multiple sclerosis were found to be more likely to report recent cannabis use compared to controls [9]. Cannabis induces neurogenesis in the hippocampus and reduces amyloid plaque formation, among other things, which can help slow the onset and progression of AD [10]. Amyotrophic lateral sclerosis (ALS) is associated with changes in the ECS, and cannabinoid receptor agonists can slow the progression of ALS by reducing inflammation. It is unclear whether endogenous cannabinoid dysregulation contributes to the development of Parkinson’s disease (PD) [11], and cannabis can influence disease onset by acting on ECS receptors and thereby. A previous study found that self-reported cannabis use was associated with an increased risk of developing autism spectrum disorder (ASD) compared to the general population [12], and may promote seizures [13]. Previous discussions of the relationship between cannabis use and migraine have been seen in the use of cannabis to treat migraine patients to relieve their symptoms, and there are a variety of views on the mechanisms of migraine occurrence that have not yet been clarified, with studies suggesting that there is an association with the ECS, and studies suggesting that cannabis use has been linked to glutamate transmission, which in turn leads to the development of migraine, which has not been consistently described [14, 15], and cannabis use is associated with an increased risk of schizophrenia (SCZ) [6, 7, 16]. Studies on the association between cannabis use and AN are scarce, but a significant genetic association between the two was found in a search [17]. Cannabis use before the age of 25 was significantly associated with increased self-reported attention-deficit/hyperactivity disorder (ADHD) symptoms in adults, and a significant genetic association, but there is no causal link [18, 19].

Cannabis, as a serious adverse lifestyle, increases the likelihood of developing neuropsychiatric disorders. Previous studies on cannabis use on neuropsychiatric disorders have only scratched the surface, and the data published to date are limited, with mixed conclusions, and mostly exploring therapeutic aspects. However, whether there is a causal relationship between the two is not clear to us, so it is crucial to better account for the relationship between cannabis use and neuropsychiatric disorders in humans.

In this context, Mendelian randomization (MR) offers a novel approach to explore the issue of causality in epidemiological studies, and because genetic factors are randomly assigned before birth, it can be used as a means to reduce confounding in the association between exposure and disease, achieving the effect of avoiding the large amount of confounding common in traditional observational epidemiology, mimicking the design of randomized controlled trials [20, 21]. Two-sample MR, an extension of the MR approach, has been widely used in studies exploring causal associations of risk factors with disease, and the present approach requires that associations of genetic instrumental variables with risk factors and genetic instrumental variables with outcomes come from different data sources [22].

Therefore, in this study, we decided to use a two-sample MR approach to assess the causal relationship between lifetime cannabis use and 10 common neuropsychiatric disorders to elucidate potential neurological causative factors and to help develop prevention strategies.

## 2. Material and Methods

### 2.1 Data Sources Description

The Genome-Wide Association Study (GWAS) summary statistics for lifetime cannabis use were derived from a GWAS meta-analysis involving a total of 184,765 participants of European ancestry from the International Cannabis Consortium, the UK Biobank, and 23andMe. To reduce the potential bias of population heterogeneity, we continued to search for and utilized data on ten common neuropsychiatric disorders among individuals of European ancestry, namely multiple sclerosis (MS), AD, ALS, ASD, SCZ, anorexia nervosa (AN), ADHD, and PD. For other detailed information regarding the exposures and outcomes, please refer to the **Supplementary materials**.

**Fig. 1.**
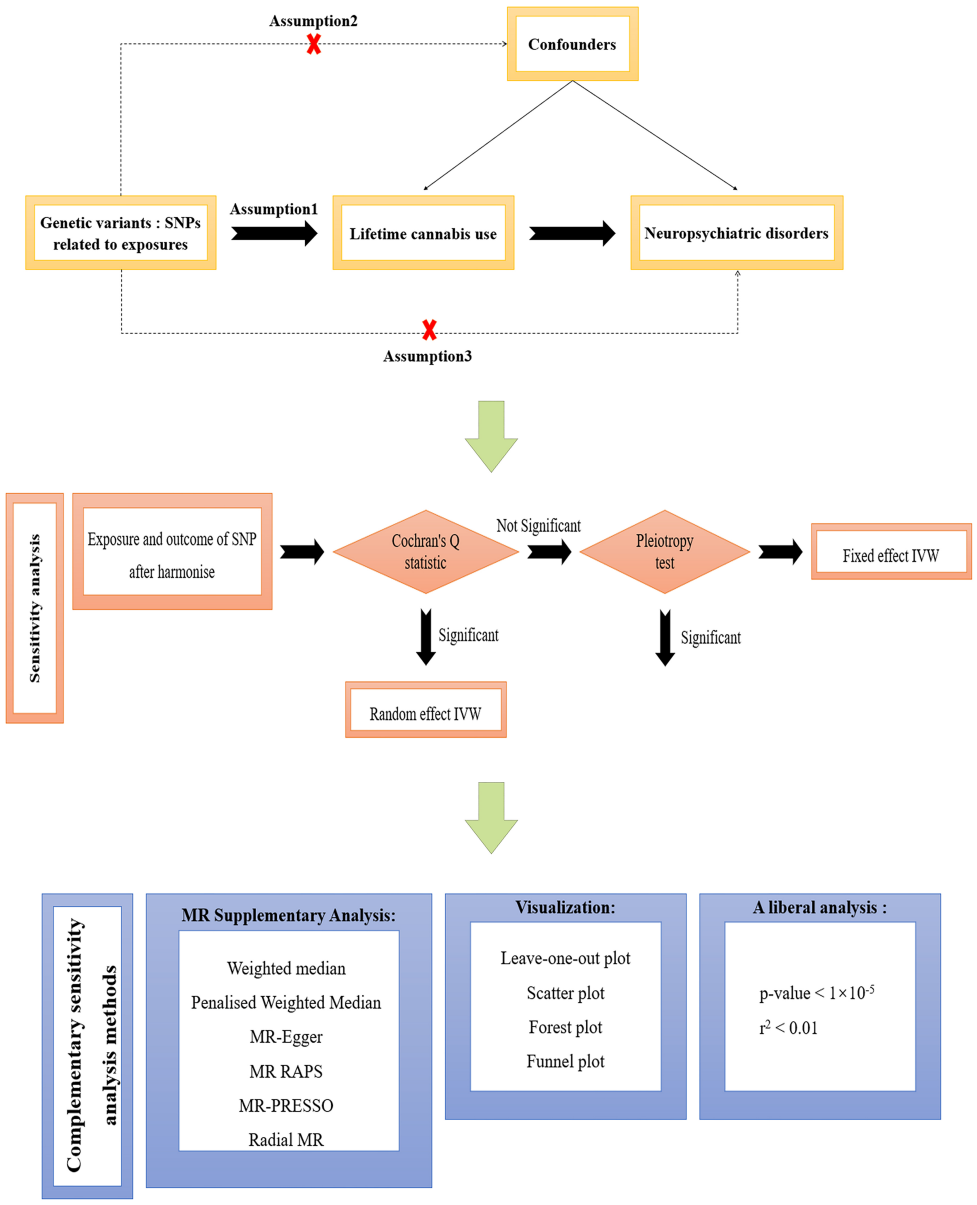
**A sketch of the overall study design in this two-sample MR study**. Lifetime cannabis use was used as the exposure and neuropsychiatric disorders were used as the outcome. Assumption 1, Assumption 2, and Assumption 3 represent the three key assumptions of Mendelian randomization. MR, Mendelian randomization study; SNP, single nucleotide polymorphism; RAPS, robust adjusted profile score; MR-PRESSO, mendelian randomization pleiotropy residual sumand outlier; IVW, inverse variance weighted.

The overall research design of this study is shown in Fig. 1, regarding an overview of the key steps of this study, a two-sample univariate MR study utilizing pooled statistics from publicly available GWAS of populations of European ancestry to assess the causal effects of lifetime marijuana use in relation to 10 common neurological disorders. Single nucleotide polymorphisms (SNPs) were selected as genetic instrumental variables (IV) by retrieving quality-controlled GWAS summary statistics and extracting SNPs that were strongly associated with lifetime cannabis use. Genetic instrumental variables should satisfy the following three key assumptions of MR: (1) Relevance: SNPs are strongly associated with lifetime cannabis use; (2) Independence: SNPs are independent of confounding factors; (3) Exclusion restriction: SNPs are not associated with neuropsychiatric disorders unless influenced through the lifetime cannabis use pathway [23]. A description of each data item is shown in **Supplementary Table 1**. Detailed information on exposure and outcome GWAS data sources are described in the **Supplementary materials**.

### 2.2 Selection of Genetic Instrumental Variables

Screen the genetic instrumental variables required for the study by the following steps. In order to satisfy the relevance assumption of MR, we first screened for SNPs significantly associated with lifetime cannabis use using a genome-wide significance threshold (*p*-value < 5 × 10^-8^) and a minor allele frequency (MAF) >0.01, and ensure that the F-statistic for each SNP is >10. To further satisfy the independence assumption of MR and to avoid SNPs being associated with confounders, a stringent linkage disequilibrium (LD) aggregation process (r^2^ < 0.001, kb = 10,000) was also set to ensure that all SNPs used as instrumental variables were mutually independently. Finally, SNPs with wrong causal direction were excluded by MR Steiger filtering test [24]. And the statistical efficacy of Mendelian randomization was calculated on the web tool (https://shiny.cnsgenomics.com/mRnd/).

### 2.3 Statistical Analysis

This study focused on the inverse variance weighted (IVW) method to analyze the causal relationship between lifetime cannabis use and common neuropsychiatric disorders. The IVW method assumes that the included SNPs are all valid, and the estimates are essentially a weighted average of the Wald ratios obtained for each SNP, and that causal effect estimates are biased once a particular SNP exhibits horizontal pleiotropy (i.e., not exposure directly affects the outcome, but through other phenotypes) [25]. However, even with up to 50% of SNPs being invalid, the weighted median approach still provides consistent estimates of causal effects [26]. In addition, we used a variety of methods robust to pleiotropy, including Penalised Weighted Median [27], MR-Egger [28] and the robust adjusted profile score (MR RAPS) [29] methods as a complement to determine the robustness of the findings. The efficacy of weighted median, Penalized Weighted Median, MR-Egger, and MR RAPS was reduced compared to IVW methods, and were only used as complementary methods in this study [30, 31].

### 2.4 Sensitivity Analysis

We performed several sensitivity analyses to further validate the MR results. First, heterogeneity was assessed by Cochran’s Q statistic obtained by the IVW method and the leave-one-out method. A Q-value <0.05 was considered to be heterogeneous. The leave-one-out method assessed whether a SNP had a significant effect on the observed results by excluding the SNP at a time. When heterogeneity was present, MR results were assessed using a random effects IVW model. Second, the presence of multiplicity in the study was determined using the intercept term and its corresponding threshold obtained by the MR-Egger method, with a non-zero intercept indicating a directional level of multiplicity [32]. The mendelian randomization pleiotropy residual sum and outlier (MR-PRESSO) method mainly uses the idea of regression, based on regression analysis of the effect of a genetic variant on an outcome against the effect of the same genetic variant on the exposure factor, and the slope of the generated regression line indicates the estimate of the causal effect of exposure on the outcome. The main function was to detect outliers and based on this, the causal effect of exposure on outcome was analyzed after removing the outliers and assessed whether there was statistical significance between the MR results of exposure on outcome before and after removing the outliers, if the results showed *p* < 0.05, the SNP needed to be removed because it indicated that the SNP was seriously affecting the MR outcome and belonged to the presence of outliers [25]. The presence of pleiotropy in the MR analysis was further assessed using radial plots and radial regression analysis, with a threshold of 0.05, the effective combination of the two methods fulfils the exclusion restriction assumption of MR. In addition, to avoid potential horizontal pleiotropy, we also performed a liberal analysis (*p*-value < 1 × 10^-5^, r^2^ < 0.01), using a loose threshold to screen for genetic instrumental variables [33].

All analyses were performed using Rstudio under version 4.2.1 (https://www.r-project.org/). The packages used in the analysis include “TwoSampleMR”, “MR-PRESSO”, “RadialMR”, “MendelianRandomization”, “forestplot”.

## 3. Results

### 3.1 Genetic Variants Selection

In our analysis, after screening, six SNPs significantly associated with lifetime cannabis use and independent of each other were finally included, which explained 1.08% of the genetic variation in exposure, corresponding to F statistics between 30.71 and 68.54 for all SNPs (Table 1), with F-statistics and general F-statistics >10 for each SNP, and the specific calculation formula is shown in the supplementary document, a result that satisfies hypothesis 1. It indicates that the SNPs included in the study have sufficient validity. The results were less susceptible to weak instrumental bias. After relaxing the threshold, 86 SNPs for lifetime cannabis use explained 10.21% of the genetic variance with F-statistics ranging from 19.58–68.54 (**Supplementary Table 2**). All analyses were obtained after MR-PRESSO method and radial plot and radial regression correction. In addition, the presence of palindromic SNPs, outliers and SNPs detected by MR Steiger filtering in the “FALSE” direction led to a variable number of IVs used in the analysis of the causal relationship between lifetime cannabis use and ten common neuropsychiatric disorders, due to the extraction of SNPs from the different outcome data in the corresponding results.

**Table 1.** **Single nucleotide polymorphisms in extracted lifetime cannabis use at *p* < 5 × 10^-8^**.

SNP	EA	OA	EAF	BETA	SE	*p*	R^2^	F-statistics
rs2875907	A	G	0.352	0.071	0.009	9.38 × 10^-17^	2.31 × 10^-3^	68.543
rs9919557	T	C	0.614	–0.055	0.009	9.94 × 10^-11^	1.43 × 10^-3^	41.716
rs10499	A	G	0.651	0.053	0.009	1.13 × 10^-9^	1.29 × 10^-3^	37.393
rs9773390	T	C	0.933	–0.171	0.029	5.66 × 10^-9^	3.69 × 10^-3^	33.988
rs10085617	A	T	0.416	0.046	0.008	2.93 × 10^-8^	1.03 × 10^-3^	30.716
rs17761723	T	C	0.346	0.047	0.009	3.24 × 10^-8^	1.01 × 10^-3^	30.966

SNP, Single nucleotide polymorphism; EA, effect allele; OA, other allele; EAF, effect allele frequency; SE, standard error; BETA, beta coefficient.

### 3.2 Analysis of Causal Effects

**Fig. 2.**
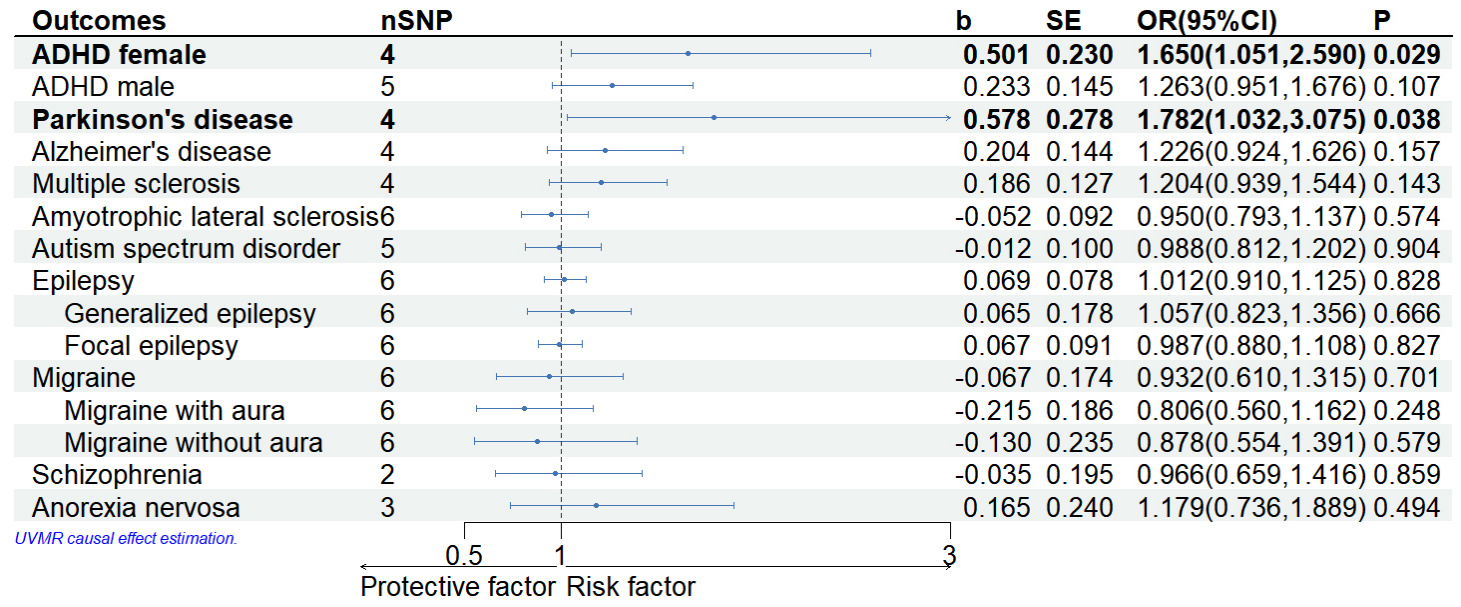
**Forest plot of results of Mendelian randomization analysis on lifetime cannabis use and Neuropsychiatric disorders**. OR, odds ratio; CI, confidence interval; ADHD, attention-deficit/hyperactivity disorder; UVMR, univariate mendelian randomization.

Under genome-wide significance conditions and after multiple testing, MR results showed that the IVW approach supported a significant causal association between genetically determined lifetime cannabis use and ADHD in female participants and PD, with cannabis use increasing the risk of disease development (ADHD in female participants: odds ratio (OR) = 1.650, 95% CI = 1.051–2.590, *p* = 0.029; PD: OR = 1.782, 95% CI = 1.032–3.075, *p* = 0.038) (Fig. 2). WM method still supports a causal relationship between lifetime cannabis use and ADHD in female participants and PD (ADHD in female participants: *p* = 0.030; PD: OR = 1.698, 95% CI = 1.052–2.740, *p *= 0.030) (**Supplementary Table 3**), at least 90% certainty to reveal a statistically significant causal relationship (**Supplementary Table 4**). The causal relationship between lifetime cannabis use and ADHD in female participants remained robust in the supplemental approach (Penalised Weighted Median: *p* = 0.024; MR RAPS: *p* = 0.031) (**Supplementary Table 3**). WM method supports increased risk of AD with cannabis use (*p* = 0.045) (**Supplementary Table 3**). Genetically predicted lifetime cannabis use was observed to be associated with an increased risk of PD and AD in the Penalised weighted median estimates (PD: *p* = 0.025; AD: *p* = 0.042) (**Supplementary Table 3**). However, the causal relationship between lifetime cannabis use and AD was not supported by the IVW and the MR RAPS methods. As we have seen, the MR results of the IVW method did not provide sufficient evidence to support a causal relationship between lifetime cannabis use and the remaining seven neuropsychiatric disorders (MS, ALS, ASD, epilepsy and migraine and its subtypes, SCZ and AN, and ADHD in male participants), i.e., *p*-value > 0.05 for MR results of multiple methods, which may indicate that lifetime cannabis use does not affect the development of these neuropsychiatric disorders. Using a liberal analysis, after a secondary analysis, the results of the IVW approach still supported a causal relationship between lifetime cannabis use and ADHD in female participants (*p* =0.011), however, not with AD and PD, and the IVW approach also provided evidence for a causal relationship between lifetime cannabis use and ASD and migraine without aura provided evidence to support a causal relationship between (ASD: *p* = 0.022; Migraine without aura: *p* = 0.004) (**Supplementary Fig. 1**).

### 3.3 Sensitivity Analysis

In sensitivity analyses, evidence of pleiotropy was shown in the causal relationship between lifetime cannabis use on migraine without aura using MR-Egger regression (migraine: intercept = 0.052, *p*_Intercept_ = 0.006; Migraine without aura: intercept = 0.063, *p*_Intercept_ = 0.033), the remaining 9 neuropsychiatric disorders were not pleiotropic. Cochran’s Q statistic results of the IVW method did not reveal potential heterogeneity among the ten neuropsychiatric disorders (Q-value >0.05) (Table 2).

**Table 2.** **Two-sample MR analysis of lifetime cannabis use for sensitivity analysis of neuropsychiatric disorders**.

Outcome	Cochran’s Q statistic	MR-Egger	MR-PRESSO		
	Q (*p*-value)	Intercept (*p*-value)	n_outliers	SNP	*p*-value of Global Test
ADHD female	4.256 (0.235)	0.032 (0.273)	0	NA	0.442
ADHD male	5.533 (0.237)	0.019 (0.496)	0	NA	0.137
PD	7.500 (0.058)	0.039 (0.705)	0	NA	0.161
AD	5.159 (0.161)	0.018 (0.342)	0	NA	0.386
MS	2.364 (0.500)	0.024 (0.197)	0	NA	0.625
ALS	1.150 (0.950)	0.009 (0.54)	0	NA	0.953
ASD	1.472 (0.832)	0.013 (0.401)	0	NA	0.819
Epilepsy	0.448 (0.799)	0.000 (0.988)	0	NA	-
Generalized epilepsy	3.586 (0.166)	0.052 (0.235)	0	NA	-
Focal epilepsy	1.220 (0.543)	0.016 (0.522)	0	NA	-
Migraine	11.044 (0.051)	0.052 (0.006)	0	NA	0.083
Migraine with aura	5.694 (0.337)	0.049 (0.080)	0	NA	0.355
Migraine without aura	8.181 (0.147)	0.063 (0.033)	0	NA	0.171
SCZ	3.383 (0.066)	0.037 (0.187)	2	rs10499/rs9919557	0.009
AN	1.097 (0.578)	0.040 (0.277)	0	NA	0.612

Abbreviations: AD, Alzheimer’s Disease; PD, Parkinson’s Disease; MS, Multiple sclerosis; ALS, Amyotrophic lateral sclerosis; ASD, Autism Spectrum Disorder; SCZ, Schizophrenia; AN, Anorexia Nervosa; NA, not applicable.

MR-PRESSO results showed the presence of two outliers SNPs in schizophrenia rs10499, rs9919557 (Table 2). After excluding outliers, we still did not find a causal relationship between lifetime cannabis use and schizophrenia. When tested using radial plots and radial regression, no outliers were found in all analyses (**Supplementary Fig. 2**). We also analyzed the MR results of the IVW method using the leave-one-out method, and the results were consistent with those of the IVW analysis after removing one SNP at a time (**Supplementary Fig. 3**). MR Steiger filtering detected SNPs with a “FALSE” orientation for 1 SNP in ADHD in female participants (rs9919557) and 2 SNPs in AN (rs17761723, rs9919557), which may be primarily associated with outcome rather than lifetime cannabis use (**Supplementary Table 5**). **Supplementary Fig. 4** and **Supplementary Fig. 5** show scatter plots and forest plots of the causal relationship between genetically predicted lifetime cannabis use and risk of neuropsychiatric disorders. We could see if there was pleiotropy in the analysis based on whether the funnel plots were symmetrical, but the number of SNPs included was too small to allow a direct assessment (**Supplementary Fig. 6**).

## 4. Discussion

The present study assessed the association between lifetime cannabis use and ten common neuropsychiatric disorders in a population of European ancestry using a two-sample MR approach. We observed evidence that lifetime cannabis use was associated with an increased risk of developing PD and ADHD in female participants, however, we found that lifetime cannabis use may act as a potential cause of AD, ASD and migraine without aura, while its causal association for MS, ALS, migraine and its migraine with aura, epilepsy and its subtypes, AN and ADHD in male participants was absent.

As mentioned earlier, the research on the association between cannabis use on ADHD is mixed. In a recent study of the causal effect of substance use on ADHD, there was no clear evidence to support a causal relationship between cannabis use and ADHD risk [18]. A recent study of the National Epidemiologic Survey on Alcohol and Related Disorders (NESARC) supported the association of cannabis use with ADHD subtypes [34], but previous studies did not consider it in terms of gender, whereas our study did find that cannabis use was associated with female patients with ADHD and not with male patients, which may be due to gender specificity. Based on previous research findings that women have higher levels of ADHD compared to men, and the use of cannabis as a form of self-medication for ADHD patients, i.e., self-medicating to alleviate the symptoms of ADHD, further provides a possible mechanistic explanation for the gender-specificity of the findings of this MR study [35, 36, 37, 38]. A search did not reveal studies related to whether cannabis use contributes to the risk of having PD, but previous studies suggest that cannabis use may have neuroprotective effects and help improve symptoms of PD [39], and our study found the possible presence of cannabis use as a risk factor for PD.

Cannabis use usually occurs in adolescence or early adulthood and may constitute an early risk factor for AD [40]. Our study did not find a causal relationship between cannabis use and AD risk. A case study showed that a 34-year-old woman with a 14-year history of cannabis abuse, diagnosed with chronic cannabis addiction, exhibiting peak epilepsy without clinical symptoms, had increasing frequency of migraine attacks during the cut-off period and a significant increase in seizure frequency after discontinuation of cannabis use [41], which is consistent with the use of cannabis as a protective factor for migraine without aura in our MR study after relaxation of the threshold of the MR study, however, we did not observe a causal relationship between cannabis use and epilepsy and its subtypes. Studies using large representative samples to examine longitudinally whether prenatal cannabis use is associated with neurodevelopment (e.g., ASD) in offspring suggest that cannabis use is a risk factor for the development of ASD [42], and in our study, the MR results obtained when using the liberal algorithm support the findings.

Previous preliminary MR analyses did not provide evidence to support a causal relationship between cannabis use and AN [17], nor did either our preliminary or secondary analyses find a causal association between the two, with results consistent with theirs. In a systematic review including 23 randomized controlled trials, cannabinoid exposure was found to cause MS relapses [43]. In a recent systematic review, including nine Randomized Controlled Trials (RCTs), it was found that there was insufficient evidence of an effect of cannabis on pain in MS patients [44]. There was no consistency between the results of the studies, and although we did not observe a causal relationship between the two, we found the emergence of cannabis as a risk factor for MS. Previous Mendelian Randomization Analysis demonstrates positive effects of schizophrenia risk and cannabis use [45]. However, after using recently obtained GWAS summary statistics showing no association between the two, using a loose threshold, we found some weak evidence but not significant indication of an association between lifetime cannabis use and schizophrenia (*p* = 0.0852). ALS is associated with changes in the endogenous cannabinoid system, cannabinoid receptor agonists may slow the progression of ALS by reducing inflammation, and there are clinical studies suggesting that cannabis may improve the symptoms of ALS [11]. Our results did not confirm a causal effect, but we observed that cannabis may be present as a protective factor for ALS.

The above discussion reveals that after relaxing the threshold, a causal association between lifetime cannabis use on certain neuropsychiatric disorders was found. However, estimates were lower for all analyses when using a relaxed threshold (*p*-value < 1 × 10^-5^) may be due to lower estimates for all analyses and weaker estimates of causality after relaxing the threshold, as these included SNPs had a weaker strength of association with lifetime cannabis use and may contain some invalid instruments [46]. Therefore, further RCTs or clinical studies are needed to validate it.

To our knowledge, this is the first study to use a two-sample MR approach to explore the causal effects between lifetime cannabis use and a variety of common neuropsychiatric disorders. First, one of the major strengths of our study is the use of a two-sample MR design, as the results of observational studies are susceptible to potential confounders and reverse causality, whereas two-sample MR analysis is an extension of the MR methodology that can utilize existing genome-wide large-sample public datasets to examine the “exposure” (as risk factor) and “outcome” (as disease) without the need to directly analyze individual-level data, which compensates for the typical shortcomings of observational studies [31, 47]. Secondly, the strength of the genetic instrumental variables included during this MR analysis was sufficiently large (F-statistics), no sample overlap was found in the GWAS data included in this study, allowing to avoid to a greater extent the influence of potential weak instrumental bias on MR results, thus the assumption of correlation of the MR instrumental variables is satisfied, further ensuring the reliability of the MR endpoints. Third, in this study we performed multiple sensitivity analyses, multiple validity tests and reverse causality tests to ensure the robustness of the MR results and to better avoid being influenced by potential confounding factors, and to ensure the accuracy of the MR endpoints.

However, there are some limitations to our study. First, the majority of participants in the GWAS summary statistics used in this study were a population of European ancestry, so extrapolation to other populations would be somewhat limited, even though the data on exposures and outcomes in this MR study are the most recent available in public databases to date. Second, despite our use of a series of sensitivity analyses and methods robust to polymorphisms, residual polymorphisms in this study may still be present. Third, the presence of individual conditions accompanied by small statistical efficacy in our analysis of the association between lifetime cannabis use and common neuropsychiatric disorders, both in a genome-wide sense and when using free analysis methods, reduces the confidence in the results, which may be due to the small sample size of the outcome and its proportion of cases. If future studies can obtain GWAS summary statistics based on larger sample sizes, they can effectively address the above issues. Fourth, since we explored the causal relationship between lifetime cannabis use and ADHD based on gender distinctions when analyzing the relationship between the two, but this is not uncommon in two-sample MR, even if the assumption of similar age and gender distributions between gene-exposure and gene-outcome is violated, MR methods can still provide evidence and thus indicate there is a causal relationship between the two [48].

## 5. Conclusions

In brief, our study supports a positive causal association between lifetime cannabis use and ADHD in female participants and PD. The potential effects of cannabis use on AD, ASD, and migraine without aura may need to be further explored, and more precise phenotypic exposures and larger sample sizes of GWAS data are needed to replicate the causal relationships. As cannabis becomes progressively legalized, the potential effects and medical use of cannabis for neuropsychiatric disorders should be considered with caution. The present study fills a knowledge gap in exploring the beneficial and harmful aspects that should be considered when using cannabis.

## Data Availability

The publicly available datasets analyzed in this study could be found in Psychiatric Genomics Consortium (https://pgc.unc.edu/for-researchers/download-results/), Open GWAS database (https://gwas.mrcieu.ac.uk/, Parkinson’s disease: ieu-b-7; Alzheimer’s disease: ieu-b-2; Multiple sclerosis: ieu-b-18; Amyotrophic lateral sclerosis: ebi-a-GCST005647; Autism Spectrum Disorder: ieu-a-1185; Epilepsy: ieu-b-8; Generalized epilepsy: ieu-b-9; Focal epilepsy: ieu-b-10; Migraine: finn-b-G6_MIGRAINE; Migraine with aura: finn-b-G6_MIGRAINE_WITH_AURA; Migraine without aura: finn-b-G6_MIGRAINE_NO_AURA; Anorexia Nervosa: ieu-a-1186) and Internation Cannabis Consortium (https://www.ru.nl/bsi/research/group-pages/substance-use-addiction-food-saf/vm-saf/genetics/international-cannabis-consortium-icc/).

## Abbreviations

SNP, single nucleotide polymorphism; OR, odds ratio; CI, confidence interval; ADHD, attention-deficit/hyperactivity disorder; AD, Alzheimer’s Disease; PD, Parkinson’s Disease; MS, Multiple sclerosis; ALS, Amyotrophic lateral sclerosis; ASD, Autism Spectrum Disorder; SCZ, Schizophrenia; AN, Anorexia Nervosa; ICC, the International Cannabis Consortium; UKBB, UK Biobank; IPDGC, the International Parkinson’s Disease Genomics Consortium; IGAP, the International Genomics of Alzheimer’s Project; ADGC, the Alzheimer’s Disease Genetics Consortium; CHARGE, the Cohorts for Heart and Aging Research in Genomic Epidemiology; EADI, the European Alzheimer Disease Initiative; GERAD, the Genetic and Environmental Research in Alzheimer Disease; IMSGC, International Multiple Sclerosis Genetics Consortium; PGC, Psychiatric Genomics Consortium; ILAE, the International League Against Epilepsy.

## Supplementary Material

Supplementary material associated with this article can be found, in the online version, at https://doi.org/10.31083/AP46108.
