# Supplementary material for: Association Between Cannabis Use and Neuropsychiatric Disorders: A Two-sample Mendelian Randomization Study
Source: Alpha Psychiatry. 2025 Aug 28;26(4):46108. doi: 10.31083/AP46108 (PMC12416058; doi:10.31083/AP46108)
Supplement: Supplementary file 1 [file 2757-8038-26-4-46108-s1.zip › Supplementary materials.pdf]

SUPPLEMENTAL MATERIALS

Supplementary Data Source Information. Detailed information of included GWAS summary statistics.
Description of the F-statistic calculation.
Figure S1. Forest plots of the results of Mendelian randomization analysis of lifetime cannabis use and neuropsychiatric disorders in liberal analysis.
Figure S2. Radial plots and radial regression: effect estimates for each individual variant.
Figure S3. A leave-one-out method for estimating single SNP Wald ratios of the effects of lifetime cannabis use on neuropsychiatric disorders.
Figure S4. Scatter plot of SNP- neuropsychiatric disorders associations vs SNP-lifetime cannabis use associations.
Figure S5. Forest plot: Mendelian randomization (MR) analyses with lifetime cannabis use as exposure and risk for neuropsychiatric disorders as outcome.
Figure S6. Funnel plot: Mendelian randomization analysis of lifetime cannabis use as exposure and risk of ten neuropsychiatric disorders as outcome.
Table S1. Single nucleotide polymorphisms in extracted lifetime cannabis use at $P < 5e-8$.
Table S2. Single nucleotide polymorphisms in extracted lifetime cannabis use at $P < 1e-5$.
Table S3. Calculating power statistics (two-sided $\alpha = 0.05$) to reflect a true causal effect ($P < 5e-8$).
Table S4. Two-sample MR analysis of the association between lifetime cannabis use and neuropsychiatric disorders.
Table S5. MR Steiger filtering results between lifetime cannabis use and neuro-psychiatric disorders.

Supplementary Data Source Information.
Detailed information of included GWAS summary statistics.

The genome-wide association study of lifetime cannabis use including 184,765 individuals of European ancestry as the exposure for this MR study [1]. The study meta-analyzed GWAS results from the International Cannabis Consortium (ICC) (N = 35,297), UK-Biobank (N = 126,785), and 23andMe (N = 22,683). Lifetime cannabis use, defined as having used any

cannabis during one's lifetime, is a heritable trait. Genotyping was performed on a variety of genotyping platforms with standard quality control checks prior to imputation. Genotype data were imputed using the 1000 Genomes Phase I release reference set49 for the ICC and 23andMe, and the Haplotype Reference Consortium reference set50 for the UK-Biobank sample. The GWAS model has been adjusted for age, sex, pedigree and genotype batch [1].

Genetic variants associated with MS from the International Multiple Sclerosis Genetics Consortium (including 47,429 cases and 68,374 controls) [2].

GWAS data for AD from the International Genomics of Alzheimer's Project (IGAP) (including 21,982 cases and 41,944 controls) were obtained from a meta-analysis of four consortia: ADGC (including 14,428 cases and 14,562 controls), CHARGE (including 2,137 cases and 13,474 controls), EADI (including 2,240 cases and 6,631 controls), and GERAD (including 3,177 cases and 7,277 controls) [3].

The GWAS summary statistics for ALS include 20,806 ALS patients and 59,804 control individuals [4].

Data on genetic variants associated with ASD, including 18,381 cases and 27,969 controls [5].

The GWAS summary statistics for epilepsy and its subtypes were obtained from The International League Against Epilepsy (ILAE) Consortium on Complex Epilepsies, which included 15,212 cases of epilepsy, 3,769 cases of focal epilepsy and 9,671 cases of generalized epilepsy and 29,677 controls[6].

The GWAS summary statistics on migraine and its subtypes were obtained from the Finngen (https://r5.finngen.fi/), which included 8547 migraines, 3,541 migraines with aura, 3,215 migraines without aura, and 176,107 controls.

Data from the Psychiatric Genomics Consortium (PGC) include the following disorders, and for the GWAS summary statistics for schizophrenia, we used the most recently published data for European pedigrees, including 53,386 patients with schizophrenia and 77,258 controls [7].

The GWAS summary statistics for AN were derived from the results of a meta-analysis of 33 datasets combining data from the Anorexia Nervosa Genetics Initiative (ANGI) and the Psychiatric Genomics Consortium's (PGC-ED) Eating Disorders Working Group, which included 16,992 cases and 55,525 controls [8].

The GWAS summary statistics for ADHD were disaggregated by gender, with females comprising 4,945 ADHD cases and 16,246 controls, and males comprising 14,154 cases and 17,948 controls [9].

The GWAS summary statistics for PD were obtained from the International Parkinson's Disease Genomics Consortium (IPDGC), which included 33,674 cases of European ancestry and 449,056 controls[10].

Description of the F-statistic calculation.

We used R^2 and F-statistics to measure the strength of IV. Each SNP was calculated using the F statistics ($F = beta^2/se^2$) and the F statistic for all SNPs was calculated using the following formula: $F = (N-K-1/K) \times R^2/(1-R^2)$, where N is the sample size of the exposure dataset, K denotes the number of SNPs extracted that are associated with exposure, and R^2 denotes the genetic variation in exposure explained by IV [11]. R^2 is calculated as follows: $R^2 = 2 \times EAF \times (1 - EAF) \times \beta^2$, where EAF represents the effect allele frequency and β denotes the estimated genetic effect on exposure [12] .

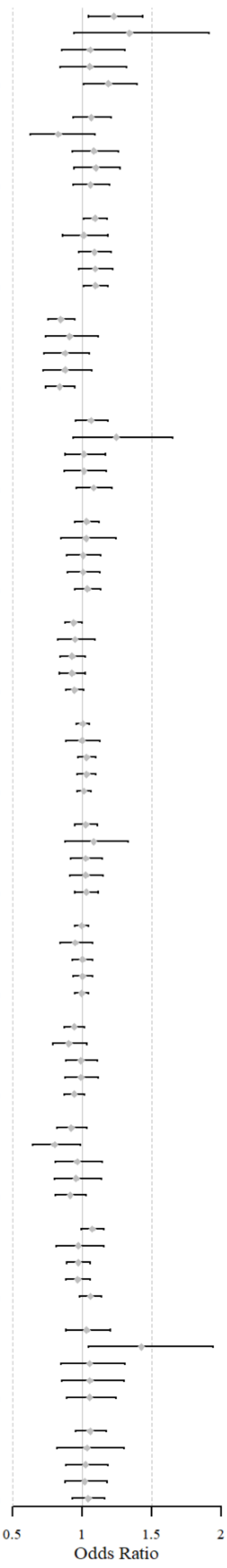

Outcome	nSNP	Method	OR(95%CI)	P
ADHD female	60	Inverse variance weighted	1.228(1.048,1.438)	1.09E-02
		MR Egger	1.341(0.941,1.913)	1.10E-01
		Weighted median	1.058(0.855,1.309)	6.03E-01
		Penalised weighted median	1.056(0.843,1.323)	6.36E-01
		Robust adjusted profile score (RAPS)	1.190(1.013,1.398)	3.46E-02
ADHD male	59	Inverse variance weighted	1.066(0.940,1.210)	3.20E-01
		MR Egger	0.830(0.629,1.095)	1.92E-01
		Weighted median	1.084(0.931,1.261)	2.99E-01
		Penalised weighted median	1.098(0.945,1.275)	2.22E-01
		Robust adjusted profile score (RAPS)	1.060(0.939,1.197)	3.42E-01
Autism Spectrum Disorder	70	Inverse variance weighted	1.093(1.013,1.180)	2.20E-02
		MR Egger	1.011(0.863,1.185)	8.88E-01
		Weighted median	1.088(0.977,1.211)	1.24E-01
		Penalised weighted median	1.094(0.981,1.221)	1.06E-01
		Robust adjusted profile score (RAPS)	1.097(1.012,1.189)	2.51E-02
Migraine without aura	80	Inverse variance weighted	0.847(0.757,0.948)	3.78E-03
		MR Egger	0.911(0.742,1.117)	3.72E-01
		Weighted median	0.877(0.730,1.054)	1.63E-01
		Penalised weighted median	0.877(0.720,1.069)	1.93E-01
		Robust adjusted profile score (RAPS)	0.838(0.739,0.951)	6.26E-03
Multiple sclerosis	59	Inverse variance weighted	1.064(0.953,1.189)	2.70E-01
		MR Egger	1.246(0.940,1.652)	1.32E-01
		Weighted median	1.013(0.878,1.168)	8.61E-01
		Penalised weighted median	1.013(0.874,1.174)	8.66E-01
		Robust adjusted profile score (RAPS)	1.083(0.963,1.218)	1.84E-01
Alzheimer's disease	70	Inverse variance weighted	1.033(0.951,1.121)	4.43E-01
		MR Egger	1.029(0.852,1.243)	7.65E-01
		Weighted median	1.007(0.893,1.134)	9.15E-01
		Penalised weighted median	1.007(0.897,1.131)	9.04E-01
		Robust adjusted profile score (RAPS)	1.039(0.950,1.136)	4.07E-01
Amyotrophic lateral sclerosis	82	Inverse variance weighted	0.940(0.879,1.004)	6.54E-02
		MR Egger	0.950(0.825,1.095)	4.81E-01
		Weighted median	0.927(0.842,1.022)	1.27E-01
		Penalised weighted median	0.928(0.839,1.026)	1.43E-01
		Robust adjusted profile score (RAPS)	0.946(0.883,1.015)	1.22E-01
epilepsy	54	Inverse variance weighted	1.006(0.960,1.054)	8.08E-01
		MR Egger	1.000(0.888,1.127)	9.99E-01
		Weighted median	1.032(0.970,1.098)	3.23E-01
		Penalised weighted median	1.032(0.969,1.099)	3.24E-01
		Robust adjusted profile score (RAPS)	1.013(0.966,1.063)	5.90E-01
generalized epilepsy	53	Inverse variance weighted	1.027(0.951,1.110)	4.91E-01
		MR Egger	1.082(0.880,1.330)	4.58E-01
		Weighted median	1.027(0.918,1.149)	6.39E-01
		Penalised weighted median	1.027(0.916,1.152)	6.49E-01
		Robust adjusted profile score (RAPS)	1.030(0.947,1.120)	4.95E-01
focal epilepsy	55	Inverse variance weighted	0.997(0.950,1.047)	9.19E-01
		MR Egger	0.952(0.842,1.076)	4.34E-01
		Weighted median	1.004(0.934,1.078)	9.20E-01
		Penalised weighted median	1.004(0.935,1.077)	9.19E-01
		Robust adjusted profile score (RAPS)	0.998(0.948,1.050)	9.31E-01
Migraine	80	Inverse variance weighted	0.944(0.876,1.016)	1.23E-01
		MR Egger	0.905(0.791,1.035)	1.50E-01
		Weighted median	0.992(0.887,1.110)	8.89E-01
		Penalised weighted median	0.992(0.882,1.116)	8.99E-01
		Robust adjusted profile score (RAPS)	0.943(0.872,1.020)	1.44E-01
Migraine with aura	80	Inverse variance weighted	0.922(0.820,1.037)	1.74E-01
		MR Egger	0.802(0.650,0.990)	4.37E-02
		Weighted median	0.965(0.811,1.148)	6.87E-01
		Penalised weighted median	0.957(0.803,1.140)	6.22E-01
		Robust adjusted profile score (RAPS)	0.914(0.812,1.029)	1.36E-01
Schizophrenia	60	Inverse variance weighted	1.074(0.998,1.156)	5.55E-02
		MR Egger	0.973(0.817,1.159)	7.58E-01
		Weighted median	0.973(0.892,1.062)	5.37E-01
		Penalised weighted median	0.968(0.887,1.057)	4.67E-01
		Robust adjusted profile score (RAPS)	1.059(0.982,1.142)	1.39E-01
Anorexia Nervosa	72	Inverse variance weighted	1.033(0.885,1.207)	6.79E-01
		MR Egger	1.426(1.046,1.942)	2.78E-02
		Weighted median	1.054(0.850,1.308)	6.30E-01
		Penalised weighted median	1.055(0.855,1.302)	6.17E-01
		Robust adjusted profile score (RAPS)	1.054(0.891,1.248)	5.37E-01
Parkinson's disease	82	Inverse variance weighted	1.058(0.953,1.175)	2.89E-01
		MR Egger	1.034(0.819,1.306)	7.79E-01
		Weighted median	1.024(0.883,1.186)	7.55E-01
		Penalised weighted median	1.019(0.879,1.181)	8.00E-01
		Robust adjusted profile score (RAPS)	1.043(0.932,1.166)	4.65E-01

Figure S1. Forest plots of the results of Mendelian randomization analysis of lifetime cannabis use and neuropsychiatric disorders in liberal analysis.

OR = probability, CI = confidence interval; nSNP, number of single nucleotide polymorphisms.

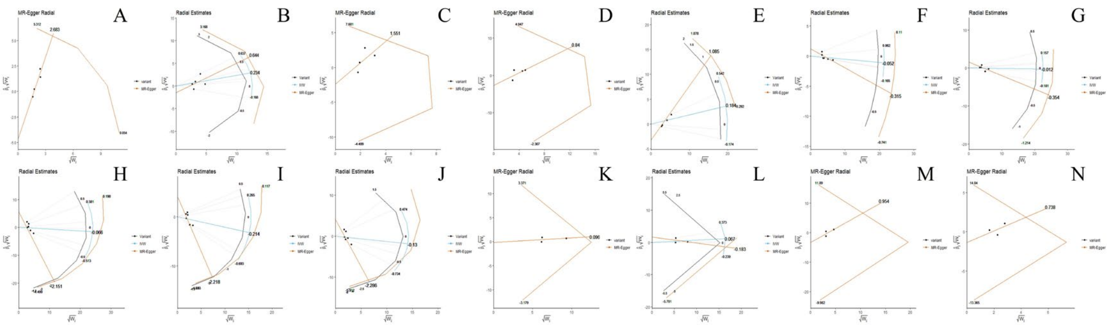

Figure S2. Radial plots and radial regression: effect estimates for each individual variant.

(A)ADHD female, (B)ADHD male, (C)Parkinson's disease, (D)Alzheimer's disease, (E)Multiple sclerosis, (F)Amyotrophic lateral sclerosis, (G) Autism spectrum disorder, (H) Epilepsy, (I) Generalized epilepsy, (J) Focal epilepsy, (K) Migraine, (L) Migraine with aura, (M) Migraine without aura, (N) Anorexia Nervosa.

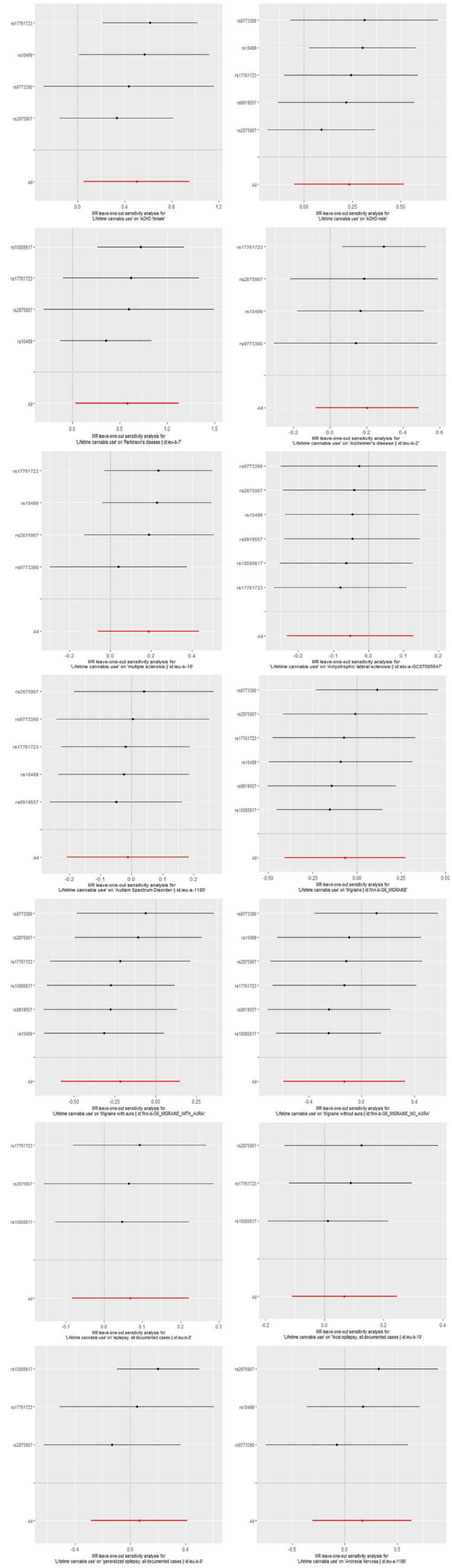

Figure S3. A leave-one-out method for estimating single SNP Wald ratios of the effects of lifetime cannabis use on

neuropsychiatric disorders. (A)ADHD female, (B)ADHD male, (C)Parkinson's disease, (D)Alzheimer's disease, (E)Multiple sclerosis, (F)Amyotrophic lateral sclerosis, (G) Autism spectrum disorder, (H) Epilepsy, (I) Generalized epilepsy, (J) Focal epilepsy, (K) Migraine, (L) Migraine with aura, (M) Migraine without aura, (N) Anorexia Nervosa.

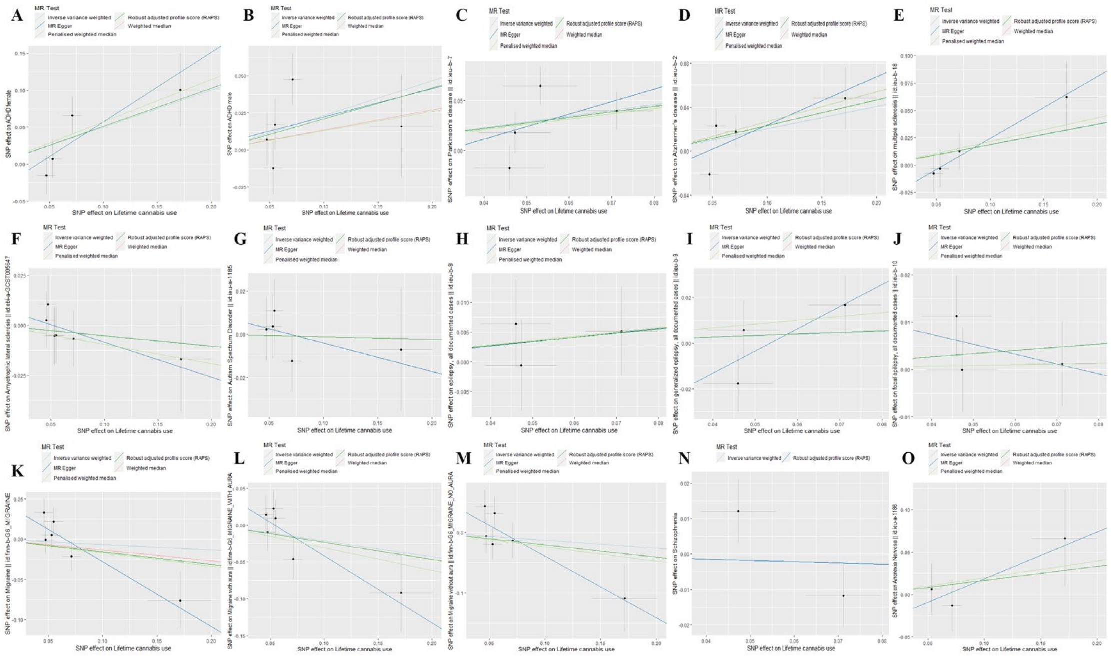

Figure S4. Scatter plot of SNP- neuropsychiatric disorders associations vs SNP-lifetime cannabis use associations. (A)ADHD female, (B)ADHD male, (C)Parkinson's disease, (D)Alzheimer's disease, (E)Multiple sclerosis, (F)Amyotrophic lateral sclerosis, (G) Autism spectrum disorder, (H) Epilepsy, (I) Generalized epilepsy, (J) Focal epilepsy, (K) Migraine, (L) Migraine with aura, (M) Migraine without aura, (N) Schizophrenia, (O) Anorexia Nervosa.

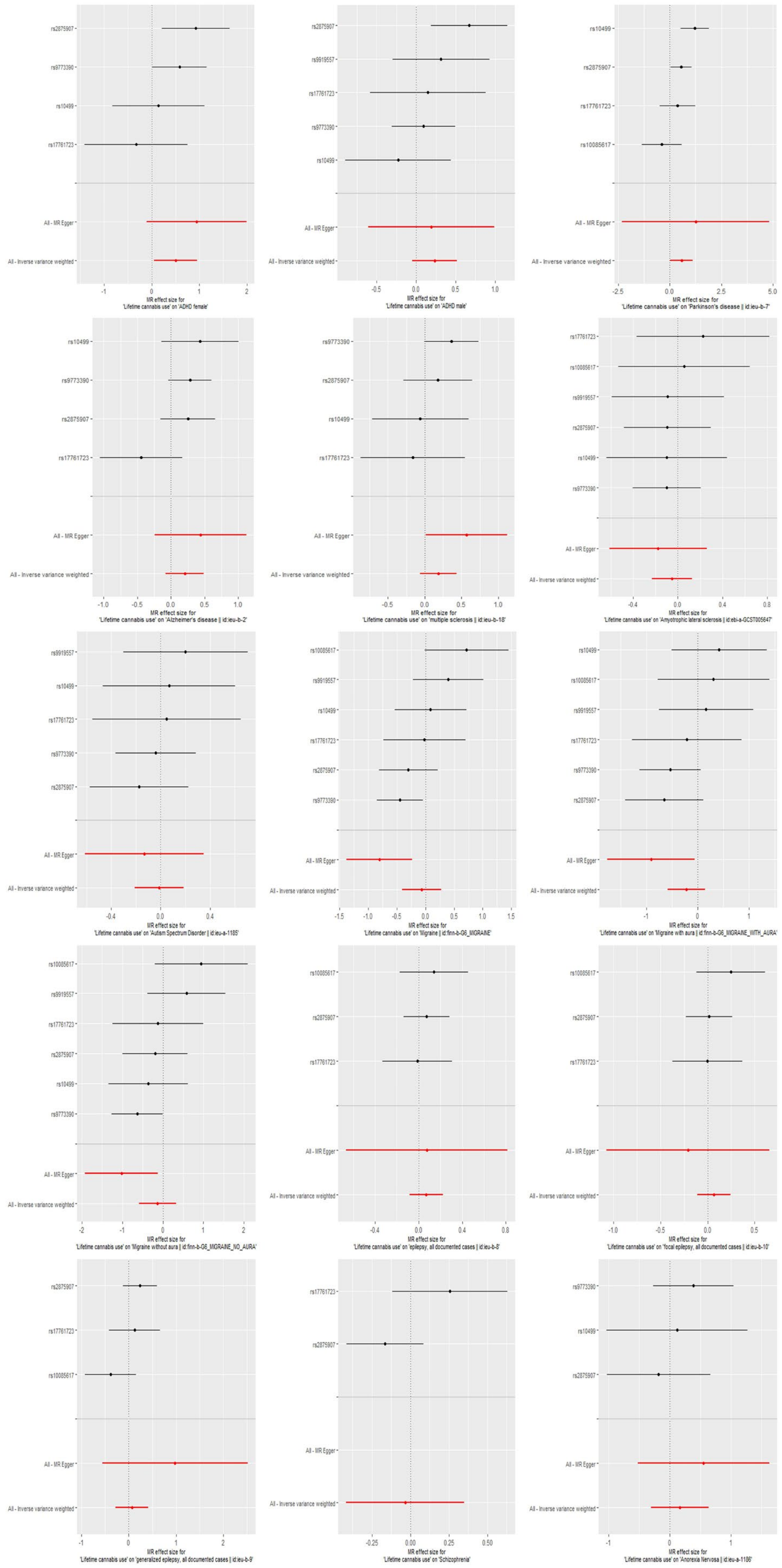

Figure S5. Forest plot: Mendelian randomization (MR) analyses with lifetime cannabis use as exposure and risk for

neuropsychiatric disorders as outcome.

(A)ADHD female, (B)ADHD male, (C)Parkinson's disease, (D)Alzheimer's disease, (E)Multiple sclerosis, (F)Amyotrophic lateral sclerosis, (G) Autism spectrum disorder, (H) Epilepsy, (I) Generalized epilepsy, (J) Focal epilepsy, (K) Migraine, (L) Migraine with aura, (M) Migraine without aura, (N) Schizophrenia, (O) Anorexia Nervosa.

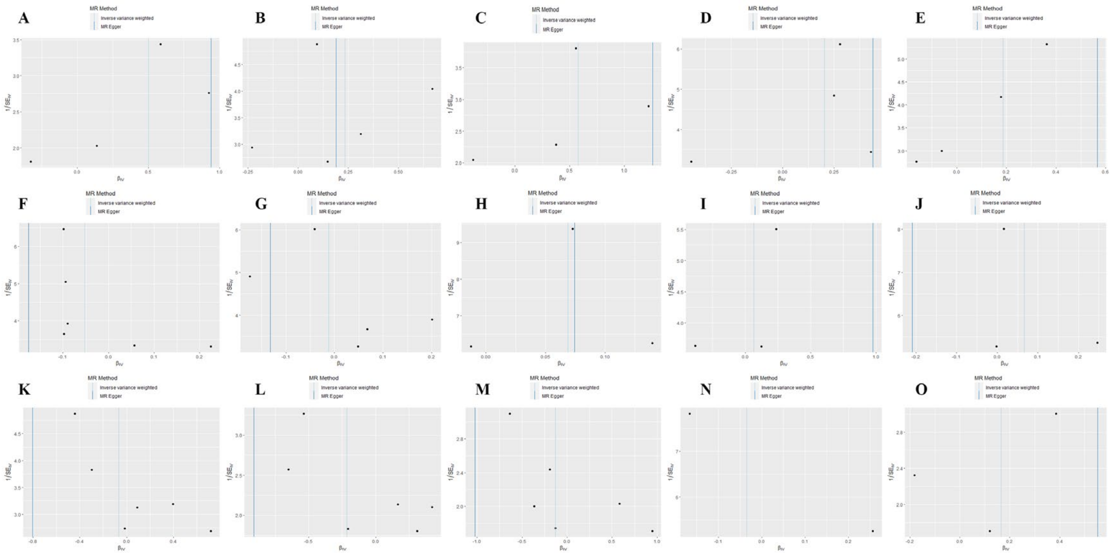

Figure S6. Funnel plot: Mendelian randomization analysis of lifetime cannabis use as exposure and risk of ten neuropsychiatric disorders as outcome. (A)ADHD female, (B)ADHD male, (C)Parkinson's disease, (D)Alzheimer's disease, (E)Multiple sclerosis, (F)Amyotrophic lateral sclerosis, (G) Autism spectrum disorder, (H) Epilepsy, (I) Generalized epilepsy, (J) Focal epilepsy, (K) Migraine, (L) Migraine with aura, (M) Migraine without aura, (N) Schizophrenia, (O) Anorexia Nervosa.

Table S1. Single nucleotide polymorphisms in extracted lifetime cannabis use at P < 5e-8.

SNP	EA	OA	EAF	BETA	SE	P	R^2	F-statistics
rs2875907	A	G	0.352	0.071	0.009	9.38E-17	2.31E-03	68.543
rs9919557	T	C	0.614	-0.055	0.009	9.94E-11	1.43E-03	41.716
rs10499	A	G	0.651	0.053	0.009	1.13E-09	1.29E-03	37.393
rs9773390	T	C	0.933	-0.171	0.029	5.66E-09	3.69E-03	33.988
rs10085617	A	T	0.416	0.046	0.008	2.93E-08	1.03E-03	30.716
rs17761723	T	C	0.346	0.047	0.009	3.24E-08	1.01E-03	30.966

Table S2. Single nucleotide polymorphisms in extracted lifetime cannabis use at P < 1e-5.

SNP	EA	OA	EAF	BETA	SE	P	R^2	F-statistics
rs2875907	A	G	0.352	0.071	0.009	9.38E-17	2.31E-03	68.543
rs9919557	T	C	0.614	-0.055	0.009	9.94E-11	1.43E-03	41.716
rs10499	A	G	0.651	0.053	0.009	1.13E-09	1.29E-03	37.393
rs9773390	T	C	0.933	-0.171	0.029	5.66E-09	3.69E-03	33.988
rs10085617	A	T	0.416	0.046	0.008	2.93E-08	1.03E-03	30.716
rs17761723	T	C	0.346	0.047	0.009	3.24E-08	1.01E-03	30.966
rs466765	A	T	0.209	0.057	0.010	5.88E-08	1.06E-03	29.514
rs11191511	T	C	0.920	-0.087	0.016	6.06E-08	1.12E-03	29.335
rs1154693	A	G	0.146	-0.063	0.012	6.92E-08	9.91E-04	28.994
rs714008	T	C	0.117	0.067	0.013	1.89E-07	9.21E-04	27.154
rs1957725	T	C	0.823	0.055	0.011	2.53E-07	8.96E-04	26.807
rs12211611	C	G	0.192	-0.054	0.010	2.55E-07	8.97E-04	26.761
rs1066339	A	G	0.168	0.147	0.029	2.72E-07	6.07E-03	26.490
rs9972422	A	G	0.709	-0.046	0.009	2.75E-07	8.80E-04	26.351
rs437021	T	C	0.459	-0.042	0.008	2.77E-07	8.84E-04	26.485
rs10008926	A	G	0.287	-0.047	0.009	2.89E-07	8.88E-04	26.223
rs7586062	C	G	0.475	0.043	0.009	3.69E-07	9.31E-04	25.830
rs576076	A	G	0.253	0.048	0.009	4.43E-07	8.61E-04	25.750
rs4308708	A	C	0.960	-0.108	0.022	5.69E-07	9.06E-04	25.046
rs114212469	T	C	0.021	0.158	0.032	6.23E-07	1.01E-03	24.780
rs1808579	T	C	0.479	0.041	0.008	6.80E-07	8.31E-04	24.757
rs9268848	A	G	0.545	-0.045	0.009	7.25E-07	1.02E-03	24.781
rs9435794	T	C	0.711	-0.046	0.009	9.20E-07	8.70E-04	23.947
rs7871607	T	G	0.988	-0.198	0.040	9.21E-07	9.08E-04	24.068
rs205723	A	G	0.414	0.041	0.008	1.03E-06	8.16E-04	23.824
rs60369116	C	G	0.025	-0.143	0.030	1.24E-06	9.79E-04	23.432
rs11902472	A	G	0.617	0.041	0.008	1.31E-06	7.75E-04	23.246
rs4377758	T	G	0.944	-0.094	0.020	1.34E-06	9.42E-04	23.386
rs79294243	T	C	0.956	-0.099	0.021	1.64E-06	8.25E-04	23.049
rs10849982	A	G	0.172	-0.052	0.011	1.76E-06	7.69E-04	22.759
rs9298105	A	T	0.452	-0.079	0.017	1.85E-06	3.09E-03	22.648
rs2059730	A	G	0.331	-0.042	0.009	1.85E-06	7.78E-04	22.671
rs146752096	T	G	0.086	0.069	0.015	1.96E-06	7.52E-04	22.710

rs1012534	A	G	0.566	0.039	0.008	2.25E-06	7.40E-04	22.389
rs1885331	T	G	0.751	0.045	0.010	2.33E-06	7.58E-04	22.438
rs4402725	A	C	0.355	0.075	0.016	2.52E-06	2.54E-03	22.233
rs12030183	T	C	0.682	0.041	0.009	2.61E-06	7.40E-04	22.026
rs2305758	T	C	0.280	0.043	0.009	2.70E-06	7.29E-04	22.299
rs78698099	A	G	0.050	-0.101	0.022	2.78E-06	9.73E-04	21.981
rs13123620	A	G	0.591	0.039	0.008	3.20E-06	7.24E-04	21.740
rs79777905	A	G	0.018	-0.172	0.037	3.32E-06	1.02E-03	21.626
rs4962265	T	C	0.292	-0.052	0.011	3.59E-06	1.11E-03	21.391
rs142789229	T	C	0.026	0.354	0.076	3.65E-06	6.34E-03	21.433
rs9855698	C	G	0.860	-0.056	0.012	3.73E-06	7.65E-04	21.372
rs1587858	T	C	0.699	-0.042	0.009	3.79E-06	7.42E-04	21.302
rs1503510	T	C	0.350	-0.040	0.009	3.94E-06	7.17E-04	21.310
rs9655332	T	G	0.423	0.067	0.015	3.95E-06	2.18E-03	21.224
rs61942416	A	G	0.078	0.077	0.017	3.99E-06	8.45E-04	21.149
rs701802	A	G	0.360	-0.041	0.009	4.14E-06	7.83E-04	20.956
rs139972333	C	G	0.997	-0.388	0.084	4.16E-06	8.41E-04	21.184
rs16839414	A	G	0.011	0.207	0.045	4.32E-06	8.91E-04	21.087
rs7513688	A	G	0.358	-0.039	0.009	4.48E-06	7.14E-04	20.989
rs2086512	A	G	0.109	0.060	0.013	4.55E-06	6.92E-04	21.019
rs184792666	T	G	0.018	-0.349	0.076	4.65E-06	4.33E-03	21.001
rs6948053	A	G	0.944	-0.086	0.019	4.66E-06	7.75E-04	20.954
rs830147	A	G	0.053	-0.170	0.037	4.73E-06	2.91E-03	20.958
rs4837004	T	C	0.660	-0.039	0.009	4.79E-06	6.97E-04	20.989
rs8110119	T	C	0.051	0.088	0.019	4.94E-06	7.44E-04	20.769
rs4147187	T	C	0.021	0.143	0.031	4.95E-06	8.46E-04	20.856
rs149434117	T	C	0.983	0.164	0.036	4.96E-06	9.06E-04	20.804
rs2335349	T	C	0.481	0.038	0.008	5.01E-06	7.10E-04	20.631
rs12949052	A	T	0.926	0.073	0.016	5.10E-06	7.32E-04	20.816
rs11749751	A	G	0.795	-0.047	0.010	5.18E-06	7.07E-04	20.872
rs6047198	T	C	0.247	-0.043	0.010	5.41E-06	6.98E-04	20.774
rs4990843	A	C	0.648	0.039	0.009	6.00E-06	6.83E-04	20.250
rs7969834	A	G	0.727	-0.041	0.009	6.58E-06	6.77E-04	20.152
rs72677792	A	G	0.068	0.134	0.030	6.59E-06	2.27E-03	20.235
rs10012797	A	T	0.917	0.068	0.015	6.63E-06	7.15E-04	20.250
rs143529057	T	C	0.011	-0.204	0.045	6.89E-06	9.02E-04	20.200
rs27307	C	G	0.426	-0.037	0.008	6.96E-06	6.80E-04	20.196
rs17294232	A	C	0.554	0.038	0.008	7.11E-06	7.02E-04	20.143
rs61997596	A	G	0.186	0.048	0.011	7.11E-06	7.10E-04	20.084
rs4717395	A	C	0.479	-0.037	0.008	7.39E-06	6.69E-04	19.922
rs9554288	T	C	0.941	0.080	0.018	7.49E-06	7.05E-04	20.099
rs79563551	T	C	0.022	-0.143	0.032	7.50E-06	8.84E-04	20.011
rs139654195	A	G	0.874	0.096	0.021	7.84E-06	2.02E-03	20.082
rs17481131	T	C	0.793	0.045	0.010	8.06E-06	6.72E-04	20.028
rs139621111	A	C	0.804	-0.088	0.020	8.15E-06	2.43E-03	19.818
rs7670670	T	C	0.224	0.044	0.010	8.18E-06	6.84E-04	20.114

rs61868490	C	G	0.133	0.058	0.013	8.41E-06	7.79E-04	19.974
rs76021452	A	G	0.024	0.142	0.032	8.55E-06	9.38E-04	19.800
rs80144387	T	G	0.070	0.086	0.019	8.76E-06	9.55E-04	19.717
rs2049824	A	C	0.533	-0.037	0.008	9.08E-06	6.63E-04	19.813
rs10849766	A	G	0.333	0.039	0.009	9.23E-06	6.79E-04	19.742
rs72798040	T	C	0.901	0.062	0.014	9.40E-06	6.95E-04	19.585
rs4445597	T	G	0.105	0.060	0.014	9.86E-06	6.81E-04	19.659

Table S3. Two-sample MR analysis of the association between lifetime cannabis use (P < 5e-8) and neuropsychiatric disorders.

**Outcomes**	**WM**	**MR RAPS**	**Penalised Weighted Median**	**MR- Egger**
	**OR (95%CI) P_value**	**OR (95%CI) P_value**	**OR (95%CI) P_value**	**OR (95%CI) P_value**
ADHD female	1.772(1.057,2.971) **0.030**	1.671(1.049,2.663) **0.031**	1.772(1.077,2.915) **0.024**	2.562(0.900,7.295) 0.220
ADHD male	1.144(0.837,1.564) 0.399	1.232(0.930,1.632) 0.146	1.138(0.826,1.568) 0.429	1.210(0.544,2.689) 0.672
PD	1.698(1.052,2.740) **0.030**	1.743(0.999,3.041) 0.051	1.698(1.067,2.700) **0.025**	3.510(0.099,124.460) 0.562
AD	1.308(1.007,1.701) **0.045**	1.262(0.973,1.637) 0.079	1.311(1.010,1.703) **0.042**	1.551(0.783,3.070) 0.335
MS	1.242(0.914,1.687) 0.165	1.206(0.925,1.572) 0.166	1.242(0.909,1.696) 0.173	1.762(1.012,3.070) 0.183
ALS	0.909(0.726,1.138) 0.404	0.949(0.788,1.144) 0.585	0.909(0.731,1.130) 0.390	0.839(0.544,1.295) 0.473
ASD	0.988(0.780,1.253) 0.922	0.988(0.806,1.210 0.906	0.988(0.781,1.250) 0.921	0.876(0.543,1.415) 0.626
Epilepsy	1.023(0.896,1.169) 0.732	1.072(0.915,1.255) 0.390	1.075(0.908,1.273) 0.399	1.077(0.515,2.255) 0.876
Generalized epilepsy	1.098(0.861,1.401) 0.450	1.071(0.769,1.490) 0.686	1.183(0.859,1.629) 0.303	2.655(0.571,12.340) 0.431
Focal epilepsy	1.007(0.869,1.168) 0.922	1.070(0.889,1.288) 0.474	1.017(0.826,1.251) 0.876	0.811(0.342,1.924) 0.718
Migraine	0.874(0.634,1.205) 0.410	0.854(0.609,1.135) 0.361	0.845(0.618,1.135) 0.291	0.448(0.253,2.135) 0.051
Migraine with aura	0.735(0.473,1.142) 0.171	0.792(0.540,1.163) 0.235	0.735(0.468,1.154) 0.181	0.403(0.172,0.942) 0.104
Migraine without aura	0.788(0.481,1.289) 0.342	0.814(0.516,1.286) 0.378	0.788(0.480,1.293) 0.345	0.359(0.146,0.880) 0.089
SCZ	-	0.964(0.706,1.318) 0.859	-	-

AN	1.217(0.720,2.055)<br>0.463	1.180(0.721,1.931)<br>0.510	1.217(0.709,2.089)<br>0.477	1.737(0.595,5.068)<br>0.497

Abbreviations: WM, weighted median; ADHD, attention-deficit/hyperactivity disorder; AD, Alzheimer's Disease; PD, Parkinson's Disease; MS, Multiple sclerosis; ALS, Amyotrophic lateral sclerosis; ASD, Autism Spectrum Disorder; SCZ, Schizophrenia; AN, Anorexia Nervosa.

Table S4. Calculating power statistics (two-sided α = 0.05) to reflect a true causal effect(P<5e-8).

Outcomes	Sample size	Proportion of cases	OR	R^2 of instrument	Power
ADHD female	21,191	0.233	1.650	0.008	0.900
ADHD male	32,102	0.441	1.263	0.010	0.550
PD	482,730	0.070	1.782	0.006	1.000
AD	63,926	0.344	1.226	0.008	0.640
MS	115,803	0.410	1.204	0.008	0.820
ALS	80,610	0.258	0.950	0.011	0.100
ASD	46,351	0.397	0.988	0.010	0.050
Epilepsy	44,889	0.339	1.071	0.004	0.070
Generalized epilepsy	33,446	0.113	1.067	0.004	0.060
Focal epilepsy	39,348	0.246	1.069	0.004	0.070
Migraine	184,654	0.046	0.935	0.011	0.090
Migraine with aura	179,648	0.020	0.806	0.011	0.220
Migraine without aura	179,322	0.018	0.878	0.011	0.110
SCZ	130,644	0.409	0.966	0.003	0.060
AN	72,517	0.234	1.179	0.007	0.390

Abbreviations: ADHD, attention-deficit/hyperactivity disorder; AD, Alzheimer's Disease; PD, Parkinson's Disease; MS, Multiple sclerosis; ALS, Amyotrophic lateral sclerosis; ASD, Autism Spectrum Disorder; SCZ, Schizophrenia; AN, Anorexia Nervosa.

Table S5. MR Steiger filtering results between lifetime cannabis use and neuropsychiatric disorders.

Outcome	SNP	r^2.exposure	r^2.outcome	Steiger_direction
ADHD female	rs10499	2.02E-04	3.66E-06	TRUE
	rs17761723	1.68E-04	1.66E-05	TRUE
	rs2875907	3.71E-04	3.08E-04	TRUE
	rs9773390	5.86E-04	1.90E-04	TRUE
	rs9919557	2.26E-04	2.36E-04	FALSE

ADHD male	rs10499	2.02E-04	1.42E-05	TRUE
	rs17761723	1.68E-04	4.87E-06	TRUE
	rs2875907	3.71E-04	2.28E-04	TRUE
	rs9773390	5.86E-04	6.47E-06	TRUE
	rs9919557	2.26E-04	3.12E-05	TRUE
PD	rs10085617	1.66E-04	1.31E-06	TRUE
	rs10499	2.02E-04	2.57E-05	TRUE
	rs17761723	1.68E-04	1.53E-06	TRUE
	rs2875907	3.71E-04	9.34E-06	TRUE
AD	rs10499	2.02E-04	3.41E-05	TRUE
	rs17761723	1.68E-04	3.15E-05	TRUE
	rs2875907	3.71E-04	2.29E-05	TRUE
	rs9773390	5.86E-04	4.60E-05	TRUE
MS	rs10499	2.02E-04	2.81E-07	TRUE
	rs17761723	1.68E-04	1.76E-06	TRUE
	rs2875907	3.71E-04	4.77E-06	TRUE
	rs9773390	5.86E-04	3.22E-05	TRUE
ALS	rs10085617	1.66E-04	4.40E-07	TRUE
	rs10499	2.02E-04	1.57E-06	TRUE
	rs17761723	1.68E-04	6.82E-06	TRUE
	rs2875907	3.71E-04	2.80E-06	TRUE
	rs9773390	5.86E-04	5.05E-06	TRUE
	rs9919557	2.26E-04	1.52E-06	TRUE
ASD	rs10499	2.02E-04	1.33E-06	TRUE
	rs17761723	1.68E-04	5.42E-07	TRUE
	rs2875907	3.71E-04	1.58E-05	TRUE
	rs9773390	5.86E-04	1.30E-06	TRUE
	rs9919557	2.26E-04	1.31E-05	TRUE
Epilepsy	rs10085617	1.66E-04	1.69E-05	TRUE
	rs17761723	1.68E-04	1.32E-07	TRUE

	rs2875907	3.71E-04	1.03E-05	TRUE
Generalized epilepsy	rs10085617	1.66E-04	5.81E-05	TRUE
	rs17761723	1.68E-04	5.84E-06	TRUE
	rs2875907	3.71E-04	5.05E-05	TRUE
Focal epilepsy	rs10085617	1.66E-04	4.43E-05	TRUE
	rs17761723	1.68E-04	2.54E-09	TRUE
	rs2875907	3.71E-04	4.50E-07	TRUE
Migraine	rs10085617	1.66E-04	2.03E-05	TRUE
	rs10499	2.02E-04	4.32E-07	TRUE
	rs17761723	1.68E-04	1.16E-08	TRUE
	rs2875907	3.71E-04	7.04E-06	TRUE
	rs9773390	5.86E-04	2.52E-05	TRUE
	rs9919557	2.26E-04	8.62E-06	TRUE
Migraine with aura	rs10085617	1.66E-04	1.75E-06	TRUE
	rs10499	2.02E-04	4.36E-06	TRUE
	rs17761723	1.68E-04	8.03E-07	TRUE
	rs2875907	3.71E-04	1.55E-05	TRUE
	rs9773390	5.86E-04	1.71E-05	TRUE
	rs9919557	2.26E-04	6.98E-07	TRUE
Migraine without aura	rs10085617	1.66E-04	1.46E-05	TRUE
	rs10499	2.02E-04	2.94E-06	TRUE
	rs17761723	1.68E-04	2.73E-07	TRUE
	rs2875907	3.71E-04	1.19E-06	TRUE
	rs9773390	5.86E-04	2.15E-05	TRUE
	rs9919557	2.26E-04	7.83E-06	TRUE
AN	rs10499	2.02E-04	2.91E-06	TRUE
	rs17761723	1.68E-04	5.22E-04	FALSE
	rs2875907	3.71E-04	1.24E-05	TRUE

	rs9773390	5.86E-04	9.29E-05	TRUE
	rs9919557	2.26E-04	2.62E-04	FALSE
SCZ	rs10499	2.02E-04	3.84E-05	TRUE
	rs17761723	1.68E-04	1.38E-05	TRUE
	rs2875907	3.71E-04	1.29E-05	TRUE
	rs9919557	2.26E-04	7.26E-05	TRUE

Abbreviations: ADHD, attention-deficit/hyperactivity disorder; AD, Alzheimer's Disease; PD, Parkinson's Disease; MS, Multiple sclerosis; ALS, Amyotrophic lateral sclerosis; ASD, Autism Spectrum Disorder; SCZ, Schizophrenia; AN, Anorexia Nervosa.

References

1. Pasman JA, Verweij KJH, Gerring Z, Stringer S, Sanchez-Roige S, Treur JL, et al. GWAS of lifetime cannabis use reveals new risk loci, genetic overlap with psychiatric traits, and a causal influence of schizophrenia. Nat Neurosci. 2018;21(9):1161-70.
2. Consortium IMSG. Multiple sclerosis genomic map implicates peripheral immune cells and microglia in susceptibility. Science. 2019;365(6460).
3. Kunkle BW, Grenier-Boley B, Sims R, Bis JC, Damotte V, Naj AC, et al. Genetic meta-analysis of diagnosed Alzheimer's disease identifies new risk loci and implicates Aβ, tau, immunity and lipid processing. Nat Genet. 2019;51(3):414-30.
4. Nicolas A, Kenna KP, Renton AE, Ticozzi N, Faghri F, Chia R, et al. Genome-wide Analyses Identify KIF5A as a Novel ALS Gene. Neuron. 2018;97(6):1268-83.e6.
5. Grove J, Ripke S, Als TD, Mattheisen M, Walters RK, Won H, et al. Identification of common genetic risk variants for autism spectrum disorder. Nat Genet. 2019;51(3):431-44.
6. Epilepsies ILAECoC. Genome-wide mega-analysis identifies 16 loci and highlights diverse biological mechanisms in the common epilepsies. Nat Commun. 2018;9(1):5269.
7. Trubetskoy V, Pardiñas AF, Qi T, Panagiotaropoulou G, Awasthi S, Bigdeli TB, et al. Mapping genomic loci implicates genes and synaptic biology in schizophrenia. Nature. 2022;604(7906):502-8.
8. Watson HJ, Yilmaz Z, Thornton LM, Hübel C, Coleman JRI, Gaspar HA, et al. Genome-wide association study identifies eight risk loci and implicates metabo-psychiatric origins for anorexia nervosa. Nat Genet. 2019;51(8):1207-14.
9. Martin J, Walters RK, Demontis D, Mattheisen M, Lee SH, Robinson E, et al. A Genetic Investigation of Sex Bias in the Prevalence of Attention-Deficit/Hyperactivity Disorder. Biol Psychiatry. 2018;83(12):1044-53.
10. Nalls MA, Blauwendraat C, Vallerga CL, Heilbron K, Bandres-Ciga S, Chang D, et al. Identification of novel risk loci, causal insights, and heritable risk for Parkinson's disease: a meta-analysis of genome-wide association studies. Lancet Neurol. 2019;18(12):1091-102.
11. Pierce BL, Ahsan H, Vanderweele TJ. Power and instrument strength requirements for Mendelian randomization studies using multiple genetic variants. Int J Epidemiol. 2011;40(3):740-52.
12. Meddens SFW, de Vlaming R, Bowers P, Burik CAP, Linnér RK, Lee C, et al. Genomic analysis of diet composition finds novel loci and associations with health and lifestyle. Mol Psychiatry. 2021;26(6):2056-69.
