# Supplementary material for: Association Between Cannabis Use and Neuropsychiatric Disorders: A Two-sample Mendelian Randomization Study
Source: Alpha Psychiatry. 2025 Aug 28;26(4):46108. doi: 10.31083/AP46108 (PMC12416058; doi:10.31083/AP46108)
Supplement: Supplementary file 1 [file 2757-8038-26-4-46108-s1.zip › Supplementary materials.docx]

**SUPPLEMENTAL MATERIALS**

**Supplementary Data Source Information.** Detailed information of included GWAS summary statistics.

**Description of the F-statistic calculation.**

**Figure S1.** Forest plots of the results of Mendelian randomization analysis of lifetime cannabis use and neuropsychiatric disorders in liberal analysis.

**Figure S2.** Radial plots and radial regression: effect estimates for each individual variant.

**Figure S3.** A leave-one-out method for estimating single SNP Wald ratios of the effects of lifetime cannabis use on neuropsychiatric disorders.

**Figure S4.** Scatter plot of SNP- neuropsychiatric disorders associations vs SNP-lifetime cannabis use associations.

**Figure S5.** Forest plot: Mendelian randomization (MR) analyses with lifetime cannabis use as exposure and risk for neuropsychiatric disorders as outcome.

**Figure S6.** Funnel plot: Mendelian randomization analysis of lifetime cannabis use as exposure and risk of ten neuropsychiatric disorders as outcome.

**Table S1.** Single nucleotide polymorphisms in extracted lifetime cannabis use at P < 5e-8.

**Table S2.** Single nucleotide polymorphisms in extracted lifetime cannabis use at P < 1e-5.

**Table S3.** Calculating power statistics (two-sided α = 0.05) to reflect a true causal effect (P < 5e-8).

**Table S4.** Two-sample MR analysis of the association between lifetime cannabis use and neuropsychiatric disorders.

**Table S5.** MR Steiger filtering results between lifetime cannabis use and neuro-psychiatric disorders.

**Supplementary Data Source Information.**

**Detailed information of included GWAS summary statistics.**

The genome-wide association study of lifetime cannabis use including 184,765 individuals of European ancestry as the exposure for this MR study [1]. The study meta-analyzed GWAS results from the International Cannabis Consortium (ICC) (N = 35,297), UK-Biobank (N = 126,785), and 23andMe (N = 22,683). Lifetime cannabis use, defined as having used any cannabis during one's lifetime, is a heritable trait. Genotyping was performed on a variety of genotyping platforms with standard quality control checks prior to imputation. Genotype data were imputed using the 1000 Genomes Phase I release reference set49 for the ICC and 23andMe, and the Haplotype Reference Consortium reference set50 for the UK-Biobank sample. The GWAS model has been adjusted for age, sex, pedigree and genotype batch [1].

Genetic variants associated with MS from the International Multiple Sclerosis Genetics Consortium (including 47,429 cases and 68,374 controls) [2].

GWAS data for AD from the [International Genomics of Alzheimer’s Project](https://consortiapedia.fastercures.org/consortia/igap/) (IGAP) (including 21,982 cases and 41,944 controls) were obtained from a meta-analysis of four consortia: ADGC (including 14,428 cases and 14,562 controls), CHARGE (including 2,137 cases and 13,474 controls), EADI (including 2,240 cases and 6,631 controls), and GERAD (including 3,177 cases and 7,277 controls) [3].

The GWAS summary statistics for ALS include 20,806 ALS patients and 59,804 control individuals [4].

Data on genetic variants associated with ASD, including 18,381 cases and 27,969 controls [5].

The GWAS summary statistics for epilepsy and its subtypes were obtained from The International League Against Epilepsy (ILAE) Consortium on Complex Epilepsies, which included 15,212 cases of epilepsy, 3,769 cases of focal epilepsy and 9,671 cases of generalized epilepsy and 29,677 controls[6].

The GWAS summary statistics on migraine and its subtypes were obtained from the Finngen (https://r5.finngen.fi/), which included 8547 migraines, 3,541 migraines with aura, 3,215 migraines without aura, and 176,107 controls.

Data from the Psychiatric Genomics Consortium (PGC) include the following disorders, and for the GWAS summary statistics for schizophrenia, we used the most recently published data for European pedigrees, including 53,386 patients with schizophrenia and 77,258 controls [7].

The GWAS summary statistics for AN were derived from the results of a meta-analysis of 33 datasets combining data from the Anorexia Nervosa Genetics Initiative (ANGI) and the Psychiatric Genomics Consortium's (PGC-ED) Eating Disorders Working Group, which included 16,992 cases and 55,525 controls [8].

The GWAS summary statistics for ADHD were disaggregated by gender, with females comprising 4,945 ADHD cases and 16,246 controls, and males comprising 14,154 cases and 17,948 controls [9].

The GWAS summary statistics for PD were obtained from the International Parkinson's Disease Genomics Consortium (IPDGC), which included 33,674 cases of European ancestry and 449,056 controls[10].

**Description of the F-statistic calculation.**

We used R^2^ and F-statistics to measure the strength of IV. Each SNP was calculated using the F statistics (F = beta^2^/se^2^) and the F statistic for all SNPs was calculated using the following formula: F = (N-K-1/K) × R^2^/(1-R^2^), where N is the sample size of the exposure dataset, K denotes the number of SNPs extracted that are associated with exposure, and R^2^ denotes the genetic variation in exposure explained by IV [11]. R^2^ is calculated as follows: R^2^ = 2 × EAF × (1 - EAF) × β^2^, where EAF represents the effect allele frequency and β denotes the estimated genetic effect on exposure [12] .

Figure S1. Forest plots of the results of Mendelian randomization analysis of lifetime cannabis use and neuropsychiatric disorders in liberal analysis.

OR = probability, CI = confidence interval; nSNP, number of single nucleotide polymorphisms.

Figure S2. Radial plots and radial regression: effect estimates for each individual variant.

(A)ADHD female, (B)ADHD male, (C)Parkinson's disease, (D)Alzheimer's disease, (E)Multiple sclerosis, (F)Amyotrophic lateral sclerosis, (G) Autism spectrum disorder, (H) Epilepsy, (I) Generalized epilepsy, (J) Focal epilepsy, (K) Migraine, (L) Migraine with aura, (M) Migraine without aura, (N) Anorexia Nervosa.

Figure S3. A leave-one-out method for estimating single SNP Wald ratios of the effects of lifetime cannabis use on neuropsychiatric disorders. (A)ADHD female, (B)ADHD male, (C)Parkinson's disease, (D)Alzheimer's disease, (E)Multiple sclerosis, (F)Amyotrophic lateral sclerosis, (G) Autism spectrum disorder, (H) Epilepsy, (I) Generalized epilepsy, (J) Focal epilepsy, (K) Migraine, (L) Migraine with aura, (M) Migraine without aura, (N) Anorexia Nervosa.

Figure S4. Scatter plot of SNP- neuropsychiatric disorders associations vs SNP-lifetime cannabis use associations. (A)ADHD female, (B)ADHD male, (C)Parkinson's disease, (D)Alzheimer's disease, (E)Multiple sclerosis, (F)Amyotrophic lateral sclerosis, (G) Autism spectrum disorder, (H) Epilepsy, (I) Generalized epilepsy, (J) Focal epilepsy, (K) Migraine, (L) Migraine with aura, (M) Migraine without aura, (N) Schizophrenia, (O) Anorexia Nervosa.

Figure S5. Forest plot: Mendelian randomization (MR) analyses with lifetime cannabis use as exposure and risk for neuropsychiatric disorders as outcome.

(A)ADHD female, (B)ADHD male, (C)Parkinson's disease, (D)Alzheimer's disease, (E)Multiple sclerosis, (F)Amyotrophic lateral sclerosis, (G) Autism spectrum disorder, (H) Epilepsy, (I) Generalized epilepsy, (J) Focal epilepsy, (K) Migraine, (L) Migraine with aura, (M) Migraine without aura, (N) Schizophrenia, (O) Anorexia Nervosa.

Figure S6. Funnel plot: Mendelian randomization analysis of lifetime cannabis use as exposure and risk of ten neuropsychiatric disorders as outcome. (A)ADHD female, (B)ADHD male, (C)Parkinson's disease, (D)Alzheimer's disease, (E)Multiple sclerosis, (F)Amyotrophic lateral sclerosis, (G) Autism spectrum disorder, (H) Epilepsy, (I) Generalized epilepsy, (J) Focal epilepsy, (K) Migraine, (L) Migraine with aura, (M) Migraine without aura, (N) Schizophrenia, (O) Anorexia Nervosa.

Table S1. Single nucleotide polymorphisms in extracted lifetime cannabis use at P < 5e-8.

| **SNP** | **EA** | **OA** | **EAF** | **BETA** | **SE** | **P** | **R^2^** | **F-statistics** |
| --- | --- | --- | --- | --- | --- | --- | --- | --- |
| rs2875907 | A | G | 0.352 | 0.071 | 0.009 | 9.38E-17 | 2.31E-03 | 68.543 |
| rs9919557 | T | C | 0.614 | -0.055 | 0.009 | 9.94E-11 | 1.43E-03 | 41.716 |
| rs10499 | A | G | 0.651 | 0.053 | 0.009 | 1.13E-09 | 1.29E-03 | 37.393 |
| rs9773390 | T | C | 0.933 | -0.171 | 0.029 | 5.66E-09 | 3.69E-03 | 33.988 |
| rs10085617 | A | T | 0.416 | 0.046 | 0.008 | 2.93E-08 | 1.03E-03 | 30.716 |
| rs17761723 | T | C | 0.346 | 0.047 | 0.009 | 3.24E-08 | 1.01E-03 | 30.966 |

Table S2. Single nucleotide polymorphisms in extracted lifetime cannabis use at P < 1e-5.

| **SNP** | **EA** | **OA** | **EAF** | **BETA** | **SE** | **P** | **R^2^** | **F-statistics** |
| --- | --- | --- | --- | --- | --- | --- | --- | --- |
| rs2875907 | A | G | 0.352 | 0.071 | 0.009 | 9.38E-17 | 2.31E-03 | 68.543 |
| rs9919557 | T | C | 0.614 | -0.055 | 0.009 | 9.94E-11 | 1.43E-03 | 41.716 |
| rs10499 | A | G | 0.651 | 0.053 | 0.009 | 1.13E-09 | 1.29E-03 | 37.393 |
| rs9773390 | T | C | 0.933 | -0.171 | 0.029 | 5.66E-09 | 3.69E-03 | 33.988 |
| rs10085617 | A | T | 0.416 | 0.046 | 0.008 | 2.93E-08 | 1.03E-03 | 30.716 |
| rs17761723 | T | C | 0.346 | 0.047 | 0.009 | 3.24E-08 | 1.01E-03 | 30.966 |
| rs466765 | A | T | 0.209 | 0.057 | 0.010 | 5.88E-08 | 1.06E-03 | 29.514 |
| rs11191511 | T | C | 0.920 | -0.087 | 0.016 | 6.06E-08 | 1.12E-03 | 29.335 |
| rs1154693 | A | G | 0.146 | -0.063 | 0.012 | 6.92E-08 | 9.91E-04 | 28.994 |
| rs714008 | T | C | 0.117 | 0.067 | 0.013 | 1.89E-07 | 9.21E-04 | 27.154 |
| rs1957725 | T | C | 0.823 | 0.055 | 0.011 | 2.53E-07 | 8.96E-04 | 26.807 |
| rs12211611 | C | G | 0.192 | -0.054 | 0.010 | 2.55E-07 | 8.97E-04 | 26.761 |
| rs1066339 | A | G | 0.168 | 0.147 | 0.029 | 2.72E-07 | 6.07E-03 | 26.490 |
| rs9972422 | A | G | 0.709 | -0.046 | 0.009 | 2.75E-07 | 8.80E-04 | 26.351 |
| rs437021 | T | C | 0.459 | -0.042 | 0.008 | 2.77E-07 | 8.84E-04 | 26.485 |
| rs10008926 | A | G | 0.287 | -0.047 | 0.009 | 2.89E-07 | 8.88E-04 | 26.223 |
| rs7586062 | C | G | 0.475 | 0.043 | 0.009 | 3.69E-07 | 9.31E-04 | 25.830 |
| rs576076 | A | G | 0.253 | 0.048 | 0.009 | 4.43E-07 | 8.61E-04 | 25.750 |
| rs4308708 | A | C | 0.960 | -0.108 | 0.022 | 5.69E-07 | 9.06E-04 | 25.046 |
| rs114212469 | T | C | 0.021 | 0.158 | 0.032 | 6.23E-07 | 1.01E-03 | 24.780 |
| rs1808579 | T | C | 0.479 | 0.041 | 0.008 | 6.80E-07 | 8.31E-04 | 24.757 |
| rs9268848 | A | G | 0.545 | -0.045 | 0.009 | 7.25E-07 | 1.02E-03 | 24.781 |
| rs9435794 | T | C | 0.711 | -0.046 | 0.009 | 9.20E-07 | 8.70E-04 | 23.947 |
| rs7871607 | T | G | 0.988 | -0.198 | 0.040 | 9.21E-07 | 9.08E-04 | 24.068 |
| rs205723 | A | G | 0.414 | 0.041 | 0.008 | 1.03E-06 | 8.16E-04 | 23.824 |
| rs60369116 | C | G | 0.025 | -0.143 | 0.030 | 1.24E-06 | 9.79E-04 | 23.432 |
| rs11902472 | A | G | 0.617 | 0.041 | 0.008 | 1.31E-06 | 7.75E-04 | 23.246 |
| rs4377758 | T | G | 0.944 | -0.094 | 0.020 | 1.34E-06 | 9.42E-04 | 23.386 |
| rs79294243 | T | C | 0.956 | -0.099 | 0.021 | 1.64E-06 | 8.25E-04 | 23.049 |
| rs10849982 | A | G | 0.172 | -0.052 | 0.011 | 1.76E-06 | 7.69E-04 | 22.759 |
| rs9298105 | A | T | 0.452 | -0.079 | 0.017 | 1.85E-06 | 3.09E-03 | 22.648 |
| rs2059730 | A | G | 0.331 | -0.042 | 0.009 | 1.85E-06 | 7.78E-04 | 22.671 |
| rs146752096 | T | G | 0.086 | 0.069 | 0.015 | 1.96E-06 | 7.52E-04 | 22.710 |
| rs1012534 | A | G | 0.566 | 0.039 | 0.008 | 2.25E-06 | 7.40E-04 | 22.389 |
| rs1885331 | T | G | 0.751 | 0.045 | 0.010 | 2.33E-06 | 7.58E-04 | 22.438 |
| rs4402725 | A | C | 0.355 | 0.075 | 0.016 | 2.52E-06 | 2.54E-03 | 22.233 |
| rs12030183 | T | C | 0.682 | 0.041 | 0.009 | 2.61E-06 | 7.40E-04 | 22.026 |
| rs2305758 | T | C | 0.280 | 0.043 | 0.009 | 2.70E-06 | 7.29E-04 | 22.299 |
| rs78698099 | A | G | 0.050 | -0.101 | 0.022 | 2.78E-06 | 9.73E-04 | 21.981 |
| rs13123620 | A | G | 0.591 | 0.039 | 0.008 | 3.20E-06 | 7.24E-04 | 21.740 |
| rs79777905 | A | G | 0.018 | -0.172 | 0.037 | 3.32E-06 | 1.02E-03 | 21.626 |
| rs4962265 | T | C | 0.292 | -0.052 | 0.011 | 3.59E-06 | 1.11E-03 | 21.391 |
| rs142789229 | T | C | 0.026 | 0.354 | 0.076 | 3.65E-06 | 6.34E-03 | 21.433 |
| rs9855698 | C | G | 0.860 | -0.056 | 0.012 | 3.73E-06 | 7.65E-04 | 21.372 |
| rs1587858 | T | C | 0.699 | -0.042 | 0.009 | 3.79E-06 | 7.42E-04 | 21.302 |
| rs1503510 | T | C | 0.350 | -0.040 | 0.009 | 3.94E-06 | 7.17E-04 | 21.310 |
| rs9655332 | T | G | 0.423 | 0.067 | 0.015 | 3.95E-06 | 2.18E-03 | 21.224 |
| rs61942416 | A | G | 0.078 | 0.077 | 0.017 | 3.99E-06 | 8.45E-04 | 21.149 |
| rs701802 | A | G | 0.360 | -0.041 | 0.009 | 4.14E-06 | 7.83E-04 | 20.956 |
| rs139972333 | C | G | 0.997 | -0.388 | 0.084 | 4.16E-06 | 8.41E-04 | 21.184 |
| rs16839414 | A | G | 0.011 | 0.207 | 0.045 | 4.32E-06 | 8.91E-04 | 21.087 |
| rs7513688 | A | G | 0.358 | -0.039 | 0.009 | 4.48E-06 | 7.14E-04 | 20.989 |
| rs2086512 | A | G | 0.109 | 0.060 | 0.013 | 4.55E-06 | 6.92E-04 | 21.019 |
| rs184792666 | T | G | 0.018 | -0.349 | 0.076 | 4.65E-06 | 4.33E-03 | 21.001 |
| rs6948053 | A | G | 0.944 | -0.086 | 0.019 | 4.66E-06 | 7.75E-04 | 20.954 |
| rs830147 | A | G | 0.053 | -0.170 | 0.037 | 4.73E-06 | 2.91E-03 | 20.958 |
| rs4837004 | T | C | 0.660 | -0.039 | 0.009 | 4.79E-06 | 6.97E-04 | 20.989 |
| rs8110119 | T | C | 0.051 | 0.088 | 0.019 | 4.94E-06 | 7.44E-04 | 20.769 |
| rs4147187 | T | C | 0.021 | 0.143 | 0.031 | 4.95E-06 | 8.46E-04 | 20.856 |
| rs149434117 | T | C | 0.983 | 0.164 | 0.036 | 4.96E-06 | 9.06E-04 | 20.804 |
| rs2335349 | T | C | 0.481 | 0.038 | 0.008 | 5.01E-06 | 7.10E-04 | 20.631 |
| rs12949052 | A | T | 0.926 | 0.073 | 0.016 | 5.10E-06 | 7.32E-04 | 20.816 |
| rs11749751 | A | G | 0.795 | -0.047 | 0.010 | 5.18E-06 | 7.07E-04 | 20.872 |
| rs6047198 | T | C | 0.247 | -0.043 | 0.010 | 5.41E-06 | 6.98E-04 | 20.774 |
| rs4990843 | A | C | 0.648 | 0.039 | 0.009 | 6.00E-06 | 6.83E-04 | 20.250 |
| rs7969834 | A | G | 0.727 | -0.041 | 0.009 | 6.58E-06 | 6.77E-04 | 20.152 |
| rs72677792 | A | G | 0.068 | 0.134 | 0.030 | 6.59E-06 | 2.27E-03 | 20.235 |
| rs10012797 | A | T | 0.917 | 0.068 | 0.015 | 6.63E-06 | 7.15E-04 | 20.250 |
| rs143529057 | T | C | 0.011 | -0.204 | 0.045 | 6.89E-06 | 9.02E-04 | 20.200 |
| rs27307 | C | G | 0.426 | -0.037 | 0.008 | 6.96E-06 | 6.80E-04 | 20.196 |
| rs17294232 | A | C | 0.554 | 0.038 | 0.008 | 7.11E-06 | 7.02E-04 | 20.143 |
| rs61997596 | A | G | 0.186 | 0.048 | 0.011 | 7.11E-06 | 7.10E-04 | 20.084 |
| rs4717395 | A | C | 0.479 | -0.037 | 0.008 | 7.39E-06 | 6.69E-04 | 19.922 |
| rs9554288 | T | C | 0.941 | 0.080 | 0.018 | 7.49E-06 | 7.05E-04 | 20.099 |
| rs79563551 | T | C | 0.022 | -0.143 | 0.032 | 7.50E-06 | 8.84E-04 | 20.011 |
| rs139654195 | A | G | 0.874 | 0.096 | 0.021 | 7.84E-06 | 2.02E-03 | 20.082 |
| rs17481131 | T | C | 0.793 | 0.045 | 0.010 | 8.06E-06 | 6.72E-04 | 20.028 |
| rs139621111 | A | C | 0.804 | -0.088 | 0.020 | 8.15E-06 | 2.43E-03 | 19.818 |
| rs7670670 | T | C | 0.224 | 0.044 | 0.010 | 8.18E-06 | 6.84E-04 | 20.114 |
| rs61868490 | C | G | 0.133 | 0.058 | 0.013 | 8.41E-06 | 7.79E-04 | 19.974 |
| rs76021452 | A | G | 0.024 | 0.142 | 0.032 | 8.55E-06 | 9.38E-04 | 19.800 |
| rs80144387 | T | G | 0.070 | 0.086 | 0.019 | 8.76E-06 | 9.55E-04 | 19.717 |
| rs2049824 | A | C | 0.533 | -0.037 | 0.008 | 9.08E-06 | 6.63E-04 | 19.813 |
| rs10849766 | A | G | 0.333 | 0.039 | 0.009 | 9.23E-06 | 6.79E-04 | 19.742 |
| rs72798040 | T | C | 0.901 | 0.062 | 0.014 | 9.40E-06 | 6.95E-04 | 19.585 |
| rs4445597 | T | G | 0.105 | 0.060 | 0.014 | 9.86E-06 | 6.81E-04 | 19.659 |

Table S3. Two-sample MR analysis of the association between lifetime cannabis use (P < 5e-8) and neuropsychiatric disorders.

| **Outcomes** | **WM** |  | **MR RAPS** |  | **Penalised Weighted Median** |  | **MR- Egger** |  |
| --- | --- | --- | --- | --- | --- | --- | --- | --- |
|  |  |  |  |  |  |  |  |  |
|  | **OR (95%CI) P_value** |  | **OR (95%CI) P_value** |  | **OR (95%CI) P_value** |  | **OR (95%CI) P_value** |  |
|  |  |  |  |  |  |  |  |  |
| ADHD female | 1.772(1.057,2.971) |  | 1.671(1.049,2.663) |  | 1.772(1.077,2.915) |  | 2.562(0.900,7.295) |  |
|  | **0.030** |  | **0.031** |  | **0.024** |  | 0.220 |  |
| ADHD male | 1.144(0.837,1.564) |  | 1.232(0.930,1.632) |  | 1.138(0.826,1.568) |  | 1.210(0.544,2.689) |  |
|  | 0.399 |  | 0.146 |  | 0.429 |  | 0.672 |  |
| PD | 1.698(1.052,2.740) |  | 1.743(0.999,3.041) |  | 1.698(1.067,2.700) |  | 3.510(0.099,124.460) |  |
|  | **0.030** |  | 0.051 |  | **0.025** |  | 0.562 |  |
| AD | 1.308(1.007,1.701) |  | 1.262(0.973,1.637) |  | 1.311(1.010,1.703) |  | 1.551(0.783,3.070) |  |
|  | **0.045** |  | 0.079 |  | **0.042** |  | 0.335 |  |
| MS | 1.242(0.914,1.687) |  | 1.206(0.925,1.572) |  | 1.242(0.909,1.696) |  | 1.762(1.012,3.070) |  |
|  | 0.165 |  | 0.166 |  | 0.173 |  | 0.183 |  |
| ALS | 0.909(0.726,1.138) |  | 0.949(0.788,1.144) |  | 0.909(0.731,1.130) |  | 0.839(0.544,1.295) |  |
|  | 0.404 |  | 0.585 |  | 0.390 |  | 0.473 |  |
| ASD | 0.988(0.780,1.253) |  | 0.988(0.806,1.210 |  | 0.988(0.781,1.250) |  | 0.876(0.543,1.415) |  |
|  | 0.922 |  | 0.906 |  | 0.921 |  | 0.626 |  |
| Epilepsy | 1.023(0.896,1.169) |  | 1.072(0.915,1.255) |  | 1.075(0.908,1.273) |  | 1.077(0.515,2.255) |  |
|  | 0.732 |  | 0.390 |  | 0.399 |  | 0.876 |  |
| Generalized epilepsy | 1.098(0.861,1.401) |  | 1.071(0.769,1.490) |  | 1.183(0.859,1.629) |  | 2.655(0.571,12.340) |  |
|  | 0.450 |  | 0.686 |  | 0.303 |  | 0.431 |  |
| Focal epilepsy | 1.007(0.869,1.168) |  | 1.070(0.889,1.288) |  | 1.017(0.826,1.251) |  | 0.811(0.342,1.924) |  |
|  | 0.922 |  | 0.474 |  | 0.876 |  | 0.718 |  |
| Migraine | 0.874(0.634,1.205) |  | 0.854(0.609,1.135) |  | 0.845(0.618,1.135) |  | 0.448(0.253,2.135) |  |
|  | 0.410 |  | 0.361 |  | 0.291 |  | 0.051 |  |
| Migraine with aura | 0.735(0.473,1.142) |  | 0.792(0.540,1.163) |  | 0.735(0.468,1.154) |  | 0.403(0.172,0.942) |  |
|  | 0.171 |  | 0.235 |  | 0.181 |  | 0.104 |  |
| Migraine without aura | 0.788(0.481,1.289) |  | 0.814(0.516,1.286) |  | 0.788(0.480,1.293) |  | 0.359(0.146,0.880) |  |
|  | 0.342 |  | 0.378 |  | 0.345 |  | 0.089 |  |
| SCZ | - |  | 0.964(0.706,1.318) |  | - |  | - |  |
|  |  |  | 0.859 |  |  |  |  |  |
| AN | 1.217(0.720,2.055) |  | 1.180(0.721,1.931) |  | 1.217(0.709,2.089) |  | 1.737(0.595,5.068) |  |
|  | 0.463 |  | 0.510 |  | 0.477 |  | 0.497 |  |

Abbreviations: WM, weighted median; ADHD, attention-deficit/hyperactivity disorder; AD, Alzheimer’s Disease; PD, Parkinson’s Disease; MS, Multiple sclerosis; ALS, Amyotrophic lateral sclerosis; ASD, Autism Spectrum Disorder; SCZ, Schizophrenia; AN, Anorexia Nervosa.

Table S4. Calculating power statistics (two-sided α = 0.05) to reflect a true causal effect(P<5e-8).

| **Outcomes** | **Sample size** | **Proportion of cases** | **OR** | **R^2^ of instrument** | **Power** |
| --- | --- | --- | --- | --- | --- |
| ADHD female | 21,191 | 0.233 | 1.650 | 0.008 | 0.900 |
| ADHD male | 32,102 | 0.441 | 1.263 | 0.010 | 0.550 |
| PD | 482,730 | 0.070 | 1.782 | 0.006 | 1.000 |
| AD | 63,926 | 0.344 | 1.226 | 0.008 | 0.640 |
| MS | 115,803 | 0.410 | 1.204 | 0.008 | 0.820 |
| ALS | 80,610 | 0.258 | 0.950 | 0.011 | 0.100 |
| ASD | 46,351 | 0.397 | 0.988 | 0.010 | 0.050 |
| Epilepsy | 44,889 | 0.339 | 1.071 | 0.004 | 0.070 |
| Generalized epilepsy | 33,446 | 0.113 | 1.067 | 0.004 | 0.060 |
| Focal epilepsy | 39,348 | 0.246 | 1.069 | 0.004 | 0.070 |
| Migraine | 184,654 | 0.046 | 0.935 | 0.011 | 0.090 |
| Migraine with aura | 179,648 | 0.020 | 0.806 | 0.011 | 0.220 |
| Migraine without aura | 179,322 | 0.018 | 0.878 | 0.011 | 0.110 |
| SCZ | 130,644 | 0.409 | 0.966 | 0.003 | 0.060 |
| AN | 72,517 | 0.234 | 1.179 | 0.007 | 0.390 |

Abbreviations: ADHD, attention-deficit/hyperactivity disorder; AD, Alzheimer’s Disease; PD, Parkinson’s Disease; MS, Multiple sclerosis; ALS, Amyotrophic lateral sclerosis; ASD, Autism Spectrum Disorder; SCZ, Schizophrenia; AN, Anorexia Nervosa.

Table S5. MR Steiger filtering results between lifetime cannabis use and neuropsychiatric disorders.

| **Outcome** | **SNP** | **r^2^.exposure** | **r^2^.outcome** | **Steiger_direction** |
| --- | --- | --- | --- | --- |
| ADHD female | rs10499 | 2.02E-04 | 3.66E-06 | TRUE |
|  | rs17761723 | 1.68E-04 | 1.66E-05 | TRUE |
|  | rs2875907 | 3.71E-04 | 3.08E-04 | TRUE |
|  | rs9773390 | 5.86E-04 | 1.90E-04 | TRUE |
|  | rs9919557 | 2.26E-04 | 2.36E-04 | FALSE |
|  |  |  |  |  |
| ADHD male | rs10499 | 2.02E-04 | 1.42E-05 | TRUE |
|  | rs17761723 | 1.68E-04 | 4.87E-06 | TRUE |
|  | rs2875907 | 3.71E-04 | 2.28E-04 | TRUE |
|  | rs9773390 | 5.86E-04 | 6.47E-06 | TRUE |
|  | rs9919557 | 2.26E-04 | 3.12E-05 | TRUE |
|  |  |  |  |  |
| PD | rs10085617 | 1.66E-04 | 1.31E-06 | TRUE |
|  | rs10499 | 2.02E-04 | 2.57E-05 | TRUE |
|  | rs17761723 | 1.68E-04 | 1.53E-06 | TRUE |
|  | rs2875907 | 3.71E-04 | 9.34E-06 | TRUE |
|  |  |  |  |  |
| AD | rs10499 | 2.02E-04 | 3.41E-05 | TRUE |
|  | rs17761723 | 1.68E-04 | 3.15E-05 | TRUE |
|  | rs2875907 | 3.71E-04 | 2.29E-05 | TRUE |
|  | rs9773390 | 5.86E-04 | 4.60E-05 | TRUE |
|  |  |  |  |  |
| MS | rs10499 | 2.02E-04 | 2.81E-07 | TRUE |
|  | rs17761723 | 1.68E-04 | 1.76E-06 | TRUE |
|  | rs2875907 | 3.71E-04 | 4.77E-06 | TRUE |
|  | rs9773390 | 5.86E-04 | 3.22E-05 | TRUE |
|  |  |  |  |  |
| ALS | rs10085617 | 1.66E-04 | 4.40E-07 | TRUE |
|  | rs10499 | 2.02E-04 | 1.57E-06 | TRUE |
|  | rs17761723 | 1.68E-04 | 6.82E-06 | TRUE |
|  | rs2875907 | 3.71E-04 | 2.80E-06 | TRUE |
|  | rs9773390 | 5.86E-04 | 5.05E-06 | TRUE |
|  | rs9919557 | 2.26E-04 | 1.52E-06 | TRUE |
|  |  |  |  |  |
| ASD | rs10499 | 2.02E-04 | 1.33E-06 | TRUE |
|  | rs17761723 | 1.68E-04 | 5.42E-07 | TRUE |
|  | rs2875907 | 3.71E-04 | 1.58E-05 | TRUE |
|  | rs9773390 | 5.86E-04 | 1.30E-06 | TRUE |
|  | rs9919557 | 2.26E-04 | 1.31E-05 | TRUE |
|  |  |  |  |  |
| Epilepsy | rs10085617 | 1.66E-04 | 1.69E-05 | TRUE |
|  | rs17761723 | 1.68E-04 | 1.32E-07 | TRUE |
|  | rs2875907 | 3.71E-04 | 1.03E-05 | TRUE |
|  |  |  |  |  |
| Generalized epilepsy | rs10085617 | 1.66E-04 | 5.81E-05 | TRUE |
|  | rs17761723 | 1.68E-04 | 5.84E-06 | TRUE |
|  | rs2875907 | 3.71E-04 | 5.05E-05 | TRUE |
|  |  |  |  |  |
| Focal epilepsy | rs10085617 | 1.66E-04 | 4.43E-05 | TRUE |
|  | rs17761723 | 1.68E-04 | 2.54E-09 | TRUE |
|  | rs2875907 | 3.71E-04 | 4.50E-07 | TRUE |
|  |  |  |  |  |
| Migraine | rs10085617 | 1.66E-04 | 2.03E-05 | TRUE |
|  | rs10499 | 2.02E-04 | 4.32E-07 | TRUE |
|  | rs17761723 | 1.68E-04 | 1.16E-08 | TRUE |
|  | rs2875907 | 3.71E-04 | 7.04E-06 | TRUE |
|  | rs9773390 | 5.86E-04 | 2.52E-05 | TRUE |
|  | rs9919557 | 2.26E-04 | 8.62E-06 | TRUE |
|  |  |  |  |  |
| Migraine with aura | rs10085617 | 1.66E-04 | 1.75E-06 | TRUE |
|  | rs10499 | 2.02E-04 | 4.36E-06 | TRUE |
|  | rs17761723 | 1.68E-04 | 8.03E-07 | TRUE |
|  | rs2875907 | 3.71E-04 | 1.55E-05 | TRUE |
|  | rs9773390 | 5.86E-04 | 1.71E-05 | TRUE |
|  | rs9919557 | 2.26E-04 | 6.98E-07 | TRUE |
|  |  |  |  |  |
| Migraine without aura | rs10085617 | 1.66E-04 | 1.46E-05 | TRUE |
|  | rs10499 | 2.02E-04 | 2.94E-06 | TRUE |
|  | rs17761723 | 1.68E-04 | 2.73E-07 | TRUE |
|  | rs2875907 | 3.71E-04 | 1.19E-06 | TRUE |
|  | rs9773390 | 5.86E-04 | 2.15E-05 | TRUE |
|  | rs9919557 | 2.26E-04 | 7.83E-06 | TRUE |
|  |  |  |  |  |
| AN | rs10499 | 2.02E-04 | 2.91E-06 | TRUE |
|  | rs17761723 | 1.68E-04 | 5.22E-04 | FALSE |
|  | rs2875907 | 3.71E-04 | 1.24E-05 | TRUE |
|  | rs9773390 | 5.86E-04 | 9.29E-05 | TRUE |
|  | rs9919557 | 2.26E-04 | 2.62E-04 | FALSE |
|  |  |  |  |  |
| SCZ | rs10499 | 2.02E-04 | 3.84E-05 | TRUE |
|  | rs17761723 | 1.68E-04 | 1.38E-05 | TRUE |
|  | rs2875907 | 3.71E-04 | 1.29E-05 | TRUE |
|  | rs9919557 | 2.26E-04 | 7.26E-05 | TRUE |

Abbreviations: ADHD, attention-deficit/hyperactivity disorder; AD, Alzheimer’s Disease; PD, Parkinson’s Disease; MS, Multiple sclerosis; ALS, Amyotrophic lateral sclerosis; ASD, Autism Spectrum Disorder; SCZ, Schizophrenia; AN, Anorexia Nervosa.

**References**

1.	Pasman JA, Verweij KJH, Gerring Z, Stringer S, Sanchez-Roige S, Treur JL, et al. GWAS of lifetime cannabis use reveals new risk loci, genetic overlap with psychiatric traits, and a causal influence of schizophrenia. Nat Neurosci. 2018;21(9):1161-70.

2.	Consortium IMSG. Multiple sclerosis genomic map implicates peripheral immune cells and microglia in susceptibility. Science. 2019;365(6460).

3.	Kunkle BW, Grenier-Boley B, Sims R, Bis JC, Damotte V, Naj AC, et al. Genetic meta-analysis of diagnosed Alzheimer's disease identifies new risk loci and implicates Aβ, tau, immunity and lipid processing. Nat Genet. 2019;51(3):414-30.

4.	Nicolas A, Kenna KP, Renton AE, Ticozzi N, Faghri F, Chia R, et al. Genome-wide Analyses Identify KIF5A as a Novel ALS Gene. Neuron. 2018;97(6):1268-83.e6.

5.	Grove J, Ripke S, Als TD, Mattheisen M, Walters RK, Won H, et al. Identification of common genetic risk variants for autism spectrum disorder. Nat Genet. 2019;51(3):431-44.

6.	Epilepsies ILAECoC. Genome-wide mega-analysis identifies 16 loci and highlights diverse biological mechanisms in the common epilepsies. Nat Commun. 2018;9(1):5269.

7.	Trubetskoy V, Pardiñas AF, Qi T, Panagiotaropoulou G, Awasthi S, Bigdeli TB, et al. Mapping genomic loci implicates genes and synaptic biology in schizophrenia. Nature. 2022;604(7906):502-8.

8.	Watson HJ, Yilmaz Z, Thornton LM, Hübel C, Coleman JRI, Gaspar HA, et al. Genome-wide association study identifies eight risk loci and implicates metabo-psychiatric origins for anorexia nervosa. Nat Genet. 2019;51(8):1207-14.

9.	Martin J, Walters RK, Demontis D, Mattheisen M, Lee SH, Robinson E, et al. A Genetic Investigation of Sex Bias in the Prevalence of Attention-Deficit/Hyperactivity Disorder. Biol Psychiatry. 2018;83(12):1044-53.

10.	Nalls MA, Blauwendraat C, Vallerga CL, Heilbron K, Bandres-Ciga S, Chang D, et al. Identification of novel risk loci, causal insights, and heritable risk for Parkinson's disease: a meta-analysis of genome-wide association studies. Lancet Neurol. 2019;18(12):1091-102.

11.	Pierce BL, Ahsan H, Vanderweele TJ. Power and instrument strength requirements for Mendelian randomization studies using multiple genetic variants. Int J Epidemiol. 2011;40(3):740-52.

12.	Meddens SFW, de Vlaming R, Bowers P, Burik CAP, Linnér RK, Lee C, et al. Genomic analysis of diet composition finds novel loci and associations with health and lifestyle. Mol Psychiatry. 2021;26(6):2056-69.
